# Best practices for the interpretation and reporting of clinical whole genome sequencing

**DOI:** 10.1038/s41525-022-00295-z

**Published:** 2022-04-08

**Authors:** Christina A. Austin-Tse, Vaidehi Jobanputra, Denise L. Perry, David Bick, Ryan J. Taft, Eric Venner, Richard A. Gibbs, Ted Young, Sarah Barnett, John W. Belmont, Nicole Boczek, Shimul Chowdhury, Katarzyna A. Ellsworth, Saurav Guha, Shashikant Kulkarni, Cherisse Marcou, Linyan Meng, David R. Murdock, Atteeq U. Rehman, Elizabeth Spiteri, Amanda Thomas-Wilson, Hutton M. Kearney, Heidi L. Rehm, Medical Genome Initiative*

**Affiliations:** 1 Center for Genomic Medicine, Massachusetts General Hospital, Boston, MA USA; 2 Laboratory for Molecular Medicine, Mass General Brigham Personalized Medicine, Cambridge, MA USA; 3 Program in Medical and Population Genetics, Broad Institute of MIT and Harvard, Cambridge, MA USA; 4 Molecular Diagnostics Laboratory, New York Genome Center, New York, NY USA; 5 Department of Pathology and Cell Biology, Columbia University Irving Medical Center, New York, NY USA; 6 Illumina Inc., San Diego, CA USA; 7 HudsonAlpha Institute for Biotechnology, Huntsville, AL USA; 8 Human Genome Sequencing Center, Baylor College of Medicine, Houston, TX USA; 9 Genome Diagnostics, Department of Paediatric Laboratory Medicine, The Hospital for Sick Children, Toronto, ON Canada; 10 Division of Laboratory Genetics and Genomics, Department of Laboratory Medicine and Pathology, Mayo Clinic, Rochester, MN USA; 11 Center for Individualized Medicine, College of Medicine, Mayo Clinic, Rochester, MN USA; 12 Rady Children’s Institute for Genomic Medicine, San Diego, CA USA; 13 Baylor Genetics and Baylor College of Medicine, Houston, TX USA; 14 Department of Molecular and Human Genetics, Baylor College of Medicine, Houston, TX USA; 15 Department of Pathology, Stanford Medicine, Stanford University, Stanford, CA USA

**Keywords:** Medical genomics, Next-generation sequencing, Genetic testing

## Abstract

Whole genome sequencing (WGS) shows promise as a first-tier diagnostic test for patients with rare genetic disorders. However, standards addressing the definition and deployment practice of a best-in-class test are lacking. To address these gaps, the Medical Genome Initiative, a consortium of leading health care and research organizations in the US and Canada, was formed to expand access to high quality clinical WGS by convening experts and publishing best practices. Here, we present best practice recommendations for the interpretation and reporting of clinical diagnostic WGS, including discussion of challenges and emerging approaches that will be critical to harness the full potential of this comprehensive test.

## Introduction

Whole genome sequencing (WGS) is emerging as a first-tier diagnostic test for rare genetic diseases^1,2^. Compared to whole exome sequencing (WES) and other molecular diagnostic tests (e.g. sequencing panels, microarrays), WGS is more comprehensive for two reasons: (i) it allows detection of a broad range of variant types in a single assay, including single nucleotide variants (SNV), small insertions and deletions, mitochondrial variants (MT), repeat expansions (RE), copy number variants (CNV) and other structural variants (SV); and (ii) it is untargeted, resulting in more uniform coverage of exonic regions^3–5^ and added coverage of intronic, intergenic and regulatory regions.

Multiple publications have demonstrated the diagnostic superiority of WGS as compared to chromosomal microarray (CMA), karyotyping, or other targeted sequencing assays^2,6–10^. While a recent meta-analysis^11^ found no significant difference in yields between WES and WGS, comparisons across cohorts, such as this one, have limited utility given the variability introduced by differing patient age groups, clinical indications, family structures, and variant types analyzed^12^. In contrast, studies comparing yields within the same cohort support diagnostic or analytical superiority of WGS^2,6,13–15^. As a result, WGS has the potential to replace most other forms of DNA-based testing.

Genome test interpretation and reporting represents a challenge to laboratories seeking to implement, or maximize the diagnostic potential of, clinical WGS. For instance, laboratories must design analytical strategies capable of efficiently prioritizing clinically relevant variation across all variant types captured by WGS (Table 1). Furthermore, to ensure that the prioritized variants are appropriately and accurately interpreted, validated, and reported, laboratories must carefully consider additional steps in the testing process, including test ordering and orthogonal confirmation.

**Table 1:** Interpretation and reporting considerations for clinically relevant variant types detectable by clinical WGS.

Variant type or category	Key interpretation and reporting considerations	Diagnostic potential^a^	Exome platform maturity^b^	Genome platform maturity^b^
Single nucleotide variants (SNV)	• Represents the largest number of variants for review, requiring phenotype-driven and genotype-driven filtering strategies (Fig. 2). • All possible consequences should be considered (e.g. review of splicing annotations, transcript-specific impacts, MNVs). • Some positions may have more than one alternate allele (multi-allelic variant)	High	High	High
Small (<150 bp) insertion and deletions (indels)	• Reviewed in the same filtering and triaging steps as SNVs. • Laboratories typically validate indels of a defined size range for review/reporting. • Some indels may be called inaccurately and may require visual inspection and/or orthogonal confirmation to resolve the variant.	High	High	High
Copy number variation (CNV)	• Filtering is based on quality, frequency, and overlap with protein-coding regions. Review of inheritance and copy number is performed during triaging. • Filtering strategies or comparisons to CNVs in variant databases should consider biological variability in CNV size and imprecision in breakpoint calls. • A depth-based plot and B-allele frequency across the genome (digital karyogram) is useful for large CNVs and aneuploidies.	High	Medium-low	Medium
Balanced and complex structural variants (SV)	Due to reduced specificity and underdeveloped appreciation of normal population variation, SV calls are primarily used for: • Detection of recurrent pathogenic balanced SVs (e.g. FVIII inversion) • Refining/characterizing CNVs detected by read depth calls • Directed search for an SV (e.g. balanced rearrangement) impacting a known region or gene of interest for the patient	Medium	Very low	Low
Runs of homozygosity (ROH)	• Extensive ROH on a single chromosome may suggest UPD. • ROH across multiple chromosomes may result from consanguinity.	Medium	High	High
Variants in regions with high homology	• Bespoke computational methods enable variant calling in regions of high homology or with known pseudogenes. • Limitations should be described in the clinical report.	Medium	Medium	Medium
Repeat expansions/ short tandem repeats (STR)	• For loci in which an approximate repeat length is returned, orthogonal characterization of the repeat length should be considered if relevant for clinical care and genetic counseling. • Visual inspection of alignments for an expanded STR call is an important QC step to provide confidence that an allele is expanded, identify interruptions, and direct orthogonal testing.	Medium	Very low	Medium
Mitochondrial variants	• Unique considerations include level of heteroplasmy, heteroplasmy threshold for disease manifestation, variable inter- and intra-familial phenotype expressivity, sample of origin (blood vs. tissue), and homology issues with NUMTs.	Medium	Capture- specific	Medium
Mosaic variants	• Due to lower depth of coverage, WGS has reduced power to detect mosaicism as compared to WES or panel testing. • Orthogonal testing may be appropriate to investigate suspected mosaic variants. • Mosaic CNVs may be called with greater sensitivity than SNVs.	Medium	Medium	Low
Polygenic risk score (PRS)	• Ancestry should be computationally assessed for use in PRS. • The majority of PRS are derived from individuals of European ancestry and correlate more poorly with risk in other ancestries. • The method of PRS calculation, including covariates, should be stated and any limitations of interpretation based on ancestry or other factors should be stated.	Low	Low^c^	Low^c^

^a^Defined here as the likelihood of identifying an etiology of disease in each variant category across a heterogeneous cohort of individuals with genetic disorders. ^b^Considers both the extent to which laboratories offer detection of each variant category and the extent to which reliable methods of detection have been developed and agreed upon by the field. ^c^While the variant detection element of the PRS calculation is reliable, the platform maturity remains low because the reporting, validity, and evidence of utility are still nascent.

*MNVs* multi-nucleotide variants, *NUMTs* nuclear mitochondrial DNA sequences, *UPD* uniparental disomy, *WES* whole exome sequencing, *WGS* whole genome sequencing.

To facilitate more widespread adoption of whole genome sequencing, the Medical Genome Initiative^16^ (MGI) formed a working group to establish best practice recommendations for the interpretation and reporting of clinical diagnostic WGS as a comprehensive test. Teleconference meetings were held over a 12-month period. Informal polling (Supplementary Note 1) was used to gain insight into the current practices of each member institution related to a multitude of topics including requisition/consent, data annotation, analysis, triage and variant curation, reporting, and reanalysis. Information obtained was used to guide the discussion and development of recommendations based on consensus among the participating laboratories. The discussions also allowed identification of areas lacking consensus and key unmet needs that, if addressed, would enable increased adoption of WGS in routine practice.

## Overview

**Fig. 1:**
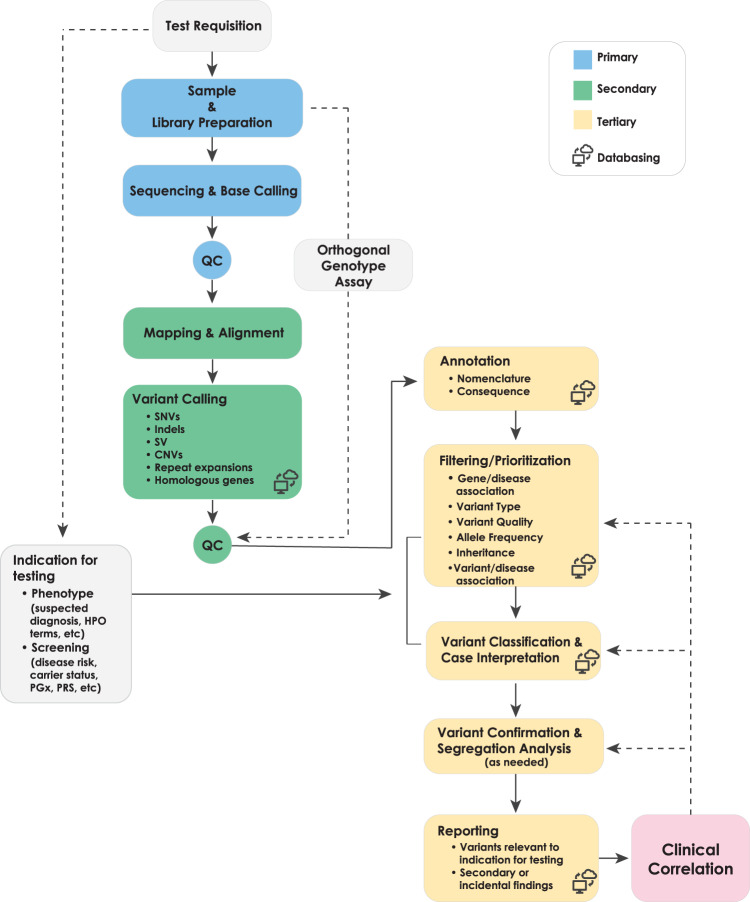
Clinical Whole Genome Sequencing Workflow. Primary WGS analysis (blue) refers to the technical production of DNA sequence data from biological samples through the process of converting raw sequencing instrument signals into nucleotides and sequence reads; secondary analysis (green) refers to the identification of DNA variants through read alignment and variant calling; and tertiary analysis (yellow) refers to adding context through variant annotation and the subsequent informatics-driven filtering, triaging, and classification of variants. Tertiary analysis also includes case interpretation, variant confirmation, segregation analysis, and reporting. While case interpretation is integrated into the laboratory process, it is important to note that clinical correlation on the part of the ordering provider is a key final step in the process and may inform additional tertiary analysis steps. Figure originally published in Marshall et al. 2020^17^.

Clinical diagnostic genomic sequencing tests can be separated into three phases of analysis: primary, secondary, and tertiary (Fig. 1). Primary analysis encompasses the technical components of the assay, including DNA extraction, library preparation, sequence generation, and preliminary data quality control (QC). Secondary analysis involves bioinformatic processes such as alignment of the raw sequence data to a genome reference, variant calling, and further data QC operations that correct for technical biases prior to analysis. Finally, tertiary analysis encompasses the interpretive steps, including annotation, filtering, prioritization, and classification of variants; case interpretation; and reporting. Whereas our first publication focused on primary and secondary analyses^17^, the focus of this manuscript is tertiary analysis. Many critical steps of tertiary analysis are reviewed below, and the supplementary materials contain numerous additional considerations. Given that our goal is to provide a comprehensive reference for WGS interpretation and reporting, this document includes many challenges and recommendations that are also relevant to WES. Genome-specific considerations are highlighted where relevant. Additionally, we acknowledge that WES and WGS are increasingly being used as a “backbone” for panel-based testing^18–20^. This document does not directly address that application, though many of the technical and interpretive aspects are also relevant to these more targeted analyses.

## Requisition/consent

The laboratory’s understanding of phenotype, family history, and types of results desired (as included in the patient consent process) drives selection of analysis strategies and reporting decisions. As a result, best practices for tertiary analysis must address the collection of this essential data on the test requisition form.

### Phenotype capture

Providing detailed phenotype information can be challenging to busy clinicians who have limited time to devote to the test ordering process. Most participating initiative institutions provide multiple options for phenotype capture on WGS test requisition forms (see Supplementary Note 2 for a sample requisition form). Clinicians most commonly opt to submit clinic notes, which typically provide adequate detail for thorough WGS analysis, especially when the note is from a relevant specialist. However, receipt of such notes requires additional effort on the part of the laboratory, who must then dedicate staff to extract salient phenotypes for use in analysis. This burden is even greater for laboratories integrated into healthcare systems, who may need to interrogate both structured and unstructured information from the electronic medical record (EMR).

For this reason, automated approaches to phenotypic data extraction from clinic notes or the full EMR have been developed for use in genomic analysis^21–23^. Preliminary investigations suggest that natural language processing (NLP) algorithms outperform manual methods in diagnostic utility of the terms selected, and that these terms can successfully prioritize reportable variants when used in genome analysis^22^. However, such systems must be optimized to data structures within individual EMR instances and their performance may vary depending on how EMRs are used by ordering physicians within their institutions. Further, NLP methods are prone to detecting a larger set of terms than manual approaches, some of which may be artifactual or include phenotypes for which the physician or patient is not requesting analysis. While NLP may be a key component of more automated analysis pipelines in the future, it has yet to see widespread implementation.

Alternative solutions include digital tools that capture detailed information in structured form. Several platforms are in current use amongst MGI laboratories, including tools that guide providers through entering structured information, such as PhenoTips^24^, and tools that automatically generate phenotype terms related to facial dysmorphism through mapping of facial features^25–27^. These tools typically structure phenotype data according to the most widely used ontology for rare disease phenotype capture: the Human Phenotype Ontology (HPO, http://www.humanphenotype-ontology.org). However, many other ontology and terminology systems are available, including OMIM (https://www.ncbi.nlm.nih.gov/omim), Disease Ontology^28^, Orphanet Rare Disease Ontology (https://www.orpha.net), the Mondo disease ontology (https://mondo.monarchinitiative.org/), SNOMED CT (https://www.nlm.nih.gov/healthit/snomedct/) and International Classification of Diseases (ICD; https://www.who.int/classifications/icd/).

Although structured phenotype information is not strictly necessary to perform high quality genome analysis, it is required for scaling of WGS testing through automation of analysis, reporting, and data sharing. Nevertheless, we caution laboratories against requiring ordering providers to submit information in primarily structured form, since placing this burden on busy clinicians may result in diminished quality and depth of phenotypic information available for analysis. Instead, laboratories may find it worthwhile to dedicate personnel effort to translating clinic notes or other unstructured information into their ontology of choice.

Regardless of the phenotype capture method used, it is recommended that phenotypic data used in genome analysis undergo review by the laboratory staff prior to the initiation of testing. Laboratories should assess whether sufficient information was provided to conduct a thorough analysis and seek clarification on unclear or conflicting information related to key phenotypes. The review process may also help to ensure that an optimal testing strategy has been selected for the patient.

### Scope of analysis and reporting

Test requisition and consent forms should clearly indicate that genetic variants relevant to any phenotype provided to the laboratory may be returned unless otherwise requested. Furthermore, in order to improve the precision of phenotype-driven analyses and variant reporting, it is recommended that the test ordering process enables physicians to specify the primary clinical question of interest. Laboratories should also consider giving patients the option to decline receiving information related to specific provided phenotypes (for example, a family history of early-onset dementia in a proband referred for an unrelated condition). However, we acknowledge that personalized reporting exclusions may be challenging. Pipelines that can dynamically integrate such preferences are desirable to accommodate these requests.

For trio or other multiple-family member sequencing approaches, consent forms should clarify how the data from auxiliary family members will be used and reported (i.e., is the parental data for a trio analyzed exclusively in the context of the proband, or might variants present only in the parents be analyzed and reported?). For reference, a sample WGS requisition form and a list of key elements of WGS consent is provided in the supplementary information (Supplementary Note 2, Supplementary Note 3).

### Secondary and incidental findings

In the course of analyzing a patient’s genome, variants expected to cause a disease unrelated to the primary indication for testing may also be identified. These unintentionally discovered variants are referred to as incidental findings (IF). Alternatively, laboratories may intentionally screen for disease-causing variants in a pre-specified set of genes that are unrelated to the indication for testing. This class of variants is referred to as secondary findings (SF)^29^. While IF and SF are generally intended to be medically actionable findings to justify their return, different approaches have been proposed to govern this process including guidance and recommendations from the ACMG, ClinGen, ESHG and other clinical and research programs^30–34^. Requisition and consent forms should clearly describe laboratory policies for SF analysis and SF and IF reporting. Ordering providers should review the laboratory’s policy ahead of offering a WGS test to inform pre-test counseling, as there are significant differences in policy and practice across laboratories^35,36^.

## Data annotation

High quality WGS interpretation is dependent upon bioinformatic data processing^17^. Here, we focus on tertiary analysis, which begins with a set of variants defined at the genomic level according to existing standards^37^. The first step of tertiary analysis is data annotation (Fig. 1), in which the predicted gene-level impact of variants is defined according to standardized nomenclature^38^ and appended with contextual information utilized in subsequent analysis steps, most notably variant prioritization and filtering.

Although annotation of WGS data typically follows a similar process as other NGS tests, no standards for NGS data annotation currently exist. Furthermore, WGS necessitates some unique considerations for annotations relevant to variant types that may not be detectable by other NGS tests (e.g. some types of structural variation). Given that data annotations determine which variants undergo expert review during the triage process, standardization could increase the consistency of WGS analyses across laboratories. To this end, we provide a list of key information utilized in the annotation pipelines of the MGI laboratories (Supplementary Data 1).

Notably, many of these annotation sources are dynamic databases. Laboratories commonly download static reference files from such databases for use in annotation pipelines. For example, a laboratory may download a file containing a current snapshot of data available in ClinVar for use in their annotation pipeline. However, new submissions are continually added to the ClinVar database, and updated downloads are available from ClinVar on a weekly basis (https://www.ncbi.nlm.nih.gov/clinvar/docs/maintenance_use/). It is therefore essential that policies are in place for both version control and regular updates to these static files. The frequency of annotation updates should take into account the pace at which new data is available from a given source as well as the burden of validation procedures required for updates to the bioinformatic pipeline^17^. Ideally, updates should directly follow the release cycles of the data sources. In the absence of infrastructure that can support continuous annotation updates from databases like ClinVar and OMIM, we recommend that annotations from these sources be updated at least quarterly.

Finally, it is worth noting that the vast majority of sequence data returned by WGS maps to noncoding and intergenic regions. Extensive functional analyses have demonstrated that variants in noncoding RNAs^39^, regulatory elements^40–42^, and deep intronic regions^43^ cause genetic disease. However, outside of previously characterized variants, the ability to interpret novel variation in noncoding regions remains limited. In order to systematically identify clinically relevant noncoding variation, resources for annotating predicted functional impacts of variation in noncoding DNA are needed. Until more resources are available, laboratories offering WGS should at least ensure that their pipelines are able to capture all known pathogenic variants in ClinVar regardless of proximity to a gene’s coding region (e.g. a known pathogenic variant that occurs in a deep intronic or promoter region).

## Analysis

Given that millions of variants are identified in an individual genome, the first step of analysis is to narrow the search space to variants with characteristics that are most likely to cause genetic disease. This selection process includes: (1) filtering, where a bioinformatic pipeline or software feature produces an output limited to variants that meet specified criteria; and (2) prioritization, where the order in which variants are presented for review is defined by specified criteria.

### Overall strategy

**Fig. 2:**
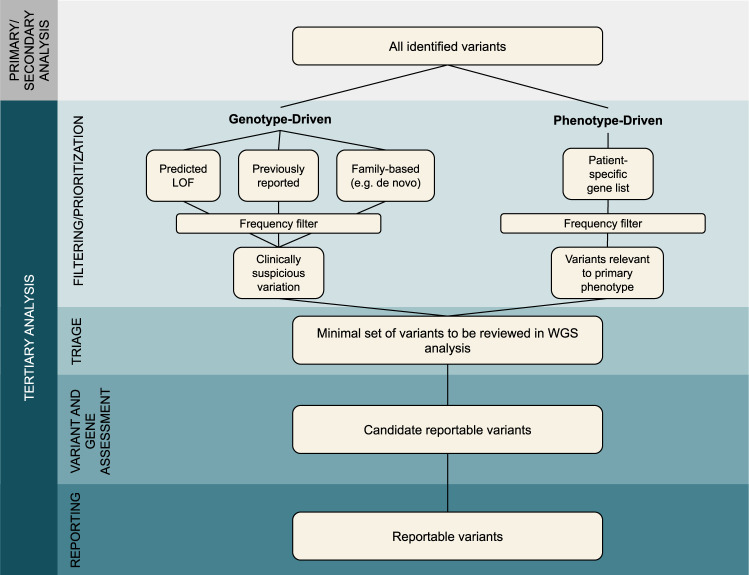
WGS analysis process, including genotype-driven and phenotype-driven approaches. Minimum necessary data filtering and prioritization approaches are shown.

Filtering and prioritization strategies for WGS analysis must strike a delicate balance between maximizing sensitivity and minimizing the number of variants requiring labor-intensive expert review. Across all analyses, encompassing various family structures (proband-only, parent-child duo, trio, and higher order family structures), different suspected modes of inheritance (e.g. a family with multiple generations of affected individuals vs an affected child with unaffected parents), and phenotypes ranging from highly specific (e.g. retinitis pigmentosa) to non-specific (e.g. intellectual disability or developmental delay), we recommend an analytical approach that incorporates both genotype-driven and phenotype-driven analyses, where appropriate (Fig. 2).

Of note, the genotype and phenotype-driven strategies defined here are primarily targeted to SNVs and small indels in the nuclear genome, which represent the majority of variants identified by WGS. However, many of the same principles apply to the analysis of additional variant types, which contribute to the diagnostic yield of this assay. Table 1 provides an overview of variant types detectable by WGS and WES as well as unique considerations for analyzing and reporting each. Example calling methods for each of these variant types are listed in Supplementary Data 2.

### Genotype-driven analysis

By screening all suspicious genetic variation regardless of disease association, the “genotype-driven” analysis is the core component of an unbiased analysis approach. This strategy facilitates the detection of: (1) unanticipated genetic diagnoses that may explain all or a portion of the patient phenotype; (2) patients with unusual presentations of established disorders (phenotypic expansion); (3) multiple genetic diagnoses in a single individual; (4) variants relevant to a secondary phenotype or family history of disease; (5) variants in novel disease genes; and (6) clinically significant incidental or secondary findings.

Genotype-driven analyses should aim to capture all variants that might have sufficient evidence to be classified as pathogenic or likely pathogenic, including: previously reported disease-causing variants, predicted loss of function (pLOF) variants (e.g. nonsense, frameshift, and essential splice sites) and, when multiple family members are sequenced, variants that are suspicious based on their inheritance pattern (e.g. *de novo* variants or biallelic rare variants in a gene associated with a recessive disorder). Automated prioritization of previously reported pathogenic variants can be challenging given the unstructured nature of the scientific literature. To aid analysts in identifying variants with known or suspected disease association, software tools may identify variants previously reported in association with any phenotype based on incorporated literature searches or database entries. ClinVar is a critical database for this purpose^44^. While variant classifications in ClinVar should not be assumed to be correct, they represent a useful tool to efficiently identify candidate variants and publications that warrant further review. In addition to freely accessible and downloadable databases like ClinVar, laboratories may choose to supplement their annotations with gene or variant-disease relationships from additional sources including subscription-based databases (see Supplementary Data 3 for databases in use at participating institutions).

In order to reduce the number of variants requiring expert review and to target variants most likely to cause genetic disease, additional filters are typically applied to genotype-driven analyses. For example, expert review may be focused on variants in or near genes that have a reported link to human disease, such as those curated by OMIM and other gene-level resources, most of which have been aggregated by the Gene Curation Coalition (thegencc.org). To further reduce the interpretive burden, laboratories also employ allele frequency cutoffs, which make use of population frequency data from reference databases such as the Genome Aggregation Database (gnomAD; https://gnomad.broadinstitute.org/) to exclude variants that are too common to cause rare genetic disease. Caution must be exercised as some cohorts in gnomAD do not represent the general population and were not screened to exclude all individuals with a genetic disease. Additionally, variants that arise from clonal hematopoiesis of indeterminate potential (CHIP)^45,46^ may falsely elevate population allele frequencies in several genes associated with germline genetic syndromes (e.g. *DNMT3A*, *ASXL1*, and *TP53*)^47^.

While reducing the number of variants requiring review is critical to the efficiency of WGS analysis, filtering criteria must ensure true pathogenic variation is not missed. For example, pathogenic founder variants, variants with reduced penetrance, or variants in genes associated with a disease of varying clinical severity may be more common in the population than the applied frequency cutoff, yet still be clinically relevant to the patient. Laboratories must design filtering approaches to account for this circumstance. For example, laboratories could review all variants classified as likely pathogenic/pathogenic in ClinVar and the laboratory’s internal knowledge base with no additional filtering criteria, or implement more permissive criteria (e.g. <5% allele frequency), to ensure these variants are returned. In addition, many laboratories maintain a list of established pathogenic variants with minor allele frequencies that are higher than their frequency cutoffs (e.g. >1%) to ensure known pathogenic high frequency variants will be reviewed. Resources for identifying these types of variants are being assembled through ClinGen^48^ and the Genetic Testing Reference Materials Coordination Program (GeT-RM, https://www.cdc.gov/labquality/get-rm/index.html). To supplement these resources, we have also provided examples of low penetrance, risk, and other high frequency variants of interest in Supplementary Data 4.

Beyond the identification of variants in well-established disease genes that match the patient phenotype, genotype-driven analysis can also be tailored to the discovery of novel disease genes. Given the rapid pace at which new genotype-phenotype correlations are discovered, reporting these findings may aid in building evidence for disease causality in a time period relevant to the patient’s care. As a result, clinical laboratories are encouraged to include analysis of genes not yet linked to disease with judicious reporting. Gene discovery analyses may prioritize *de novo* and/or pLOF variants in highly constrained genes based on gnomAD constraint scores as well as biallelic pLOF variants in genes that are devoid of homozygous LOF variants in gnomAD.

### Phenotype-driven analysis

In most cases, the genotype-driven analysis should be supplemented with additional “phenotype-driven” analyses, particularly if there are specific genes that are highly relevant to the patient’s phenotype. Phenotype-driven analyses allow for the comprehensive review of potentially relevant variants that may not meet the criteria defined in the genotype-driven analysis approach described above (e.g. novel missense variants in dominant genes). Variants identified exclusively by phenotype-driven analyses are more likely to be classified as benign or variants of uncertain significance given that they have no prior reports of pathogenicity, no *de novo* occurrence, and no predicted LOF impact, all of which would surface through genotype-driven analyses. Nevertheless, they may still meet criteria for reporting if located in a gene strongly associated with the patient’s phenotype (see reporting section below). Some nonspecific or highly genetically heterogeneous phenotypes (e.g. developmental delay and autism) are less likely to benefit from phenotype-driven analysis strategies. Therefore, the decision regarding the appropriateness of this approach is at the discretion of the analysis team.

When performing phenotype-driven analyses, laboratories must have a mechanism for defining the genes of interest for a given phenotype. Automated phenotype-driven analyses, such as those integrated into commercially available genomic analysis platforms, typically depend on structured patient phenotype data (e.g. HPO terms) to prioritize variants found in genes relevant to the patient’s phenotype. While automated methods have clear benefits in terms of efficiency, their performance varies depending on the algorithms and gene-phenotype association sources used.

Alternatively, laboratories may manually curate a list of relevant genes that can be used to prioritize variants for downstream analysis. There are multiple available sources of gene-disease association information (see Supplementary Data 5). Of note, there is no single source from which all relevant genes can reliably be mined. As a result, the curation of gene-disease associations from multiple databases is likely to produce a more comprehensive list. Furthermore, caution should be exercised as idiosyncrasies in search functionality and gene-disease annotations can lead to missing critical genes (e.g. if a database associates the *GJB2* gene exclusively with “hearing loss”, a query using the term “deafness” may not return the gene). Given multiple potential sources of error in the curation process, many MGI laboratories incorporate a QC review step for the gene lists used in each case.

When evaluating whether a given gene should be included on a curated gene list, it is advisable to consider the possibilities of phenotypic expansion, variable expressivity, age-related penetrance, or the possibilities that additional syndromic features of disease have not been clinically recognized in the patient. Furthermore, given that WGS is poised to identify relatively new or ultra-rare conditions, laboratories are encouraged to consider both well-established genes and those with more preliminary evidence for disease causality when curating gene lists for phenotype-driven analyses.

It is recommended that laboratories generate and adhere to a policy for the frequency at which curated gene lists will be reviewed and updated. If phenotype-driven analyses are performed in parallel with genotype-driven strategies that are able to capture suspicious variants in novel disease genes, we support current ACMG recommendations for updating gene panels at 6 month intervals^49^. To improve scalability of gene panel curation and maintenance, community efforts are underway to develop shared resources for defining and updating gene panels^50^.

### Copy number variants

Current variant callers can achieve high sensitivity for calling both small and large CNVs ranging in size from exon-level events to those involving multiple megabases of genomic material and hundreds of genes^51–53^. While it is possible to annotate and filter CNVs based on criteria such as quality metrics, population frequency, intersection with protein coding genes, and inheritance, automated filtering of CNVs is less straightforward for several reasons, including potential imprecision of breakpoint calling and heterogeneous (dissimilar) CNVs represented in aggregate population frequencies. Variant callers may even represent the same event with slightly different breakpoints in each family member. To avoid missing clinically relevant variants due to breakpoint imprecision, laboratories are advised to include a buffer (e.g. 1 kb) on breakpoints when filtering based on intersection with protein-coding regions. Furthermore, allele frequency thresholds for common CNVs should be carefully validated for use in filtering, with consideration of both similarity of breakpoints and equivalent copy number states. Given these complexities, MGI laboratories more commonly apply population frequency and inheritance assessments during manual interpretive review steps rather than automated filtering.

### Balanced and complex structural variants

Balanced and complex structural variants (SVs) are an important class of variation in human disease that is detectable by WGS, but difficult to detect by other NGS-based methods, including WES. However, due to reduced specificity from current callers and an emerging, but currently underdeveloped appreciation of normal population SV variation^54^, a comprehensive genome-wide search through the many thousands of SV calls is not currently feasible for routine clinical WGS. At present, SV calls are primarily used as a secondary approach to refine and characterize CNVs detected by read depth calls. Alternatively, if the laboratory is suspicious of, or directed to, a region or gene of interest, SVs associated with balanced rearrangements can be identified with targeted bioinformatic strategies. With appropriate validation, and a sufficiently narrow clinically-directed search, balanced rearrangements may be identified and reported by WGS. However, laboratories should clearly communicate the scope of SV review and limitations in sensitivity^55^.

Visualizing CNV data both as a depth-based plot and B-allele frequency across the whole genome as a “digital karyogram”^52^ is also useful to evaluate large structural rearrangements and aneuploidies, and to appreciate classic cytogenetic mechanisms (e.g. predicted unbalanced translocations, recombinant inversions^56^), which may inform significant recurrence risk for parents with balanced rearrangements.

### Mitochondrial variants

Mitochondrial DNA (mtDNA) variation can also be detected with high sensitivity by WGS^57^. Prioritization and curation of mtDNA variants is similar to that previously discussed for SNVs with some exceptions. Mitochondrial variants can exist in a continuous allele fraction range reflecting heteroplasmy. With the appropriate validation studies, the level of heteroplasmy for variants of interest can be determined and is essential in causal interpretation. The clinical phenotype of a patient, including severity, may correlate with the heteroplasmy level of a variant within an affected tissue (i.e., threshold effect) and heteroplasmy levels observed in tissues such as blood may not represent those in an affected tissue. Heteroplasmy levels may also differ between related individuals and the segregation of heteroplasmy levels should be considered as part of genome interpretation^58^. In addition to heteroplasmy, the mtDNA haplogroup of a variant may also impact disease severity and should be assessed. Resources including Mitomap^59^, MSeqDR^60^, HmtVar^61^, HelixMTdb (Helix.com/MITO), and gnomAD v3.1^57^ provide a source of mitochondrial-specific allele frequencies, in silico tools, haplogroup information and literature.

An additional consideration when assessing analytical validity of a mitochondrial variant is the presence of nuclear sequences of mitochondrial origin (NuMTs), which can negatively impact the sensitivity of mitochondrial variant calling^62^. Annotation of NuMTs is important to eliminate artifacts from the interpretive pipeline.

### Short tandem repeats

The capability to identify short tandem repeat (STR) expansions along with SNVs, indels, and CNVs, enhances the diagnostic potential of WGS as compared to WES and may reduce the need for additional stand-alone testing (e.g. for Fragile X, myotonic dystrophy, or Friedreich ataxia). Computational methods capable of detecting expanded STR loci from PCR-free WGS data (e.g. ExpansionHunter^63^) provide an avenue to detect and report on clinically relevant STRs^64,65^; however, existing algorithms have limitations in terms of sensitivity and specificity^63,66,67^.

Genes included in STR analysis may vary by laboratory and the validation performed, and the laboratory should provide information on the loci evaluated, and limitations of performance relative to stand-alone testing for these expansions. Additional considerations for analysis of STRs are provided in Table 1.

### Automated analysis

Automated analysis tools aim to computationally prioritize variants suspected to be pathogenic based on structured phenotype terms provided by the user. These methods have been reported to increase diagnostic yield and the efficiency of analysis^22,68,69^. However, their sensitivity and specificity remain insufficient to replace the role of a human analyst^70,71^. Instead, these tools should be considered as a support system to supplement the analysis strategies outlined above, particularly in the prioritization of suspicious variants. Results from automated analyses should be carefully assessed by a human analyst prior to consideration for reporting.

### Validation of filtering and prioritization strategies

Prior to launch, laboratories should validate their pipelines, including automated or manual gene list assembly approaches, to demonstrate that all “reportable” variants for a given set of cases are successfully filtered and/or prioritized by their defined analysis procedures. To do this, laboratories may be able to assemble a set of clinical test cases with known causative variants from de-identified samples sequenced by other methodologies at their institution. Additionally, laboratories should consider generating synthetic controls^72^ or fabricated BAM files tailored to identify potential weak spots in the analysis process. The CDC Get-RM Project recently collaborated with ClinGen to identify variants that are either major contributors to disease or represent technically challenging variants to detect in clinically significant genes, which can be used to help ensure important variants are adequately detected^73^.

## Case and variant interpretation

**Fig. 3:**
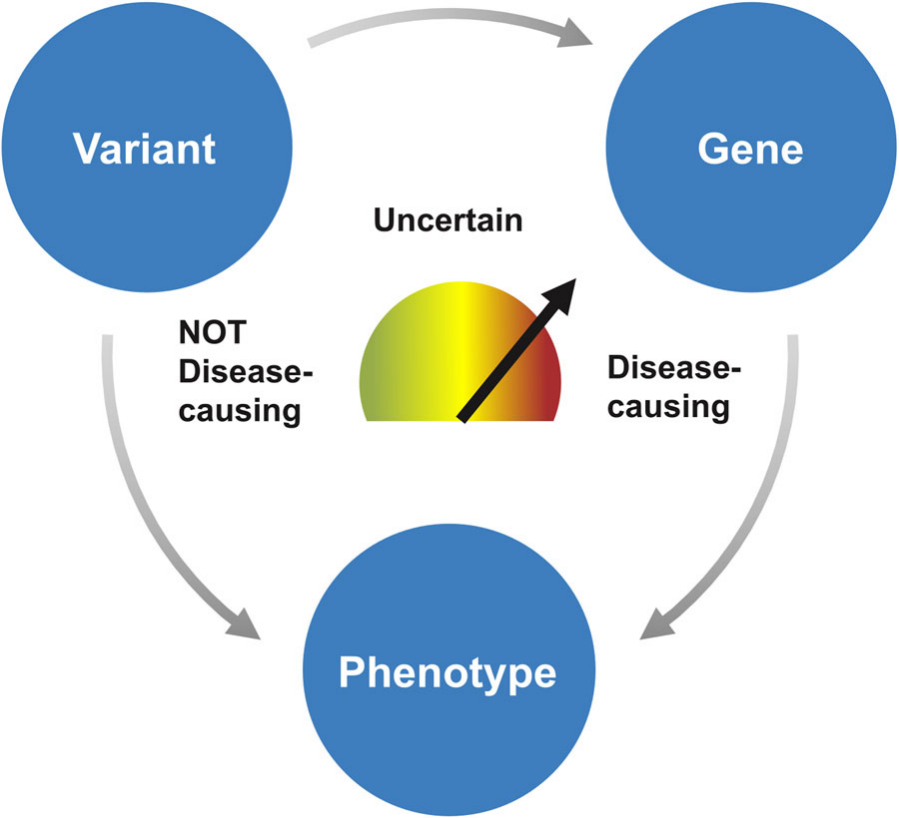
Triage review decision-making process. Variant, gene, and phenotype information must be simultaneously integrated to determine which variants should be nominated as potentially reportable.

MGI laboratories reported averages of 66–314 sequence variants returned by filtering strategies applied to a typical case (Supplementary Data 6) prior to contextualization by the patient’s phenotype. The initial variant review process, in which laboratories initiate decision-making regarding the relevance of variants to the indication(s) for testing, is described here as “triage.” The primary goal of triage is to further narrow the results of the analysis strategies described above to those variants that may meet reporting criteria. Triage involves a high-level, expert review of the available evidence supporting or refuting the variant’s pathogenicity and the disease association for the impacted gene(s) as well as relevance to the patient’s phenotype (Fig. 3). Analysts approach each variant by considering a series of questions, including:

- What gene(s) does the variant impact?
- Is the variant expected to impact the function of the gene(s) and if so, does it cause a human phenotype?
- How well does the variant or gene’s disease association match that of the patient?
- Has this particular variant (or this variant type, e.g. LOF variants) been shown to cause a phenotype?
- Is the variant returnable as a secondary or incidental finding?

Amongst the participating MGI laboratories, case analysis takes an average of 7.3 h per case (Supplementary Data 6), sometimes split across multiple highly trained staff members (primarily PhD-level variant analysts or genetic counselors trained in genomic analysis, Supplementary Fig. 1). As such, analysis represents the most time consuming and costly step of WGS analysis.

For each variant, the analyst must evaluate information that reflects a spectrum of prior knowledge of association between a human genetic disease, the variant, and the gene. Thus, triage does not lend itself easily to automation. However, triage efficiency can be substantially influenced by the software used to support analysis. The ideal infrastructure can integrate results for all variant types detectable by WGS, which is particularly valuable for the recognition of compound heterozygous variation across variant types (e.g. a nonsense variant in trans with a multi-exon deletion). The ideal infrastructure will also present a reviewer with all of the pertinent annotations and scientific literature for a given variant and the gene(s) it impacts, thereby minimizing the need to seek out information from external data sources and allowing the analyst to quickly synthesize the information and weigh the potential relevance to the patient’s indication for testing. Additionally, software systems should provide a means for reviewers to record comments and provide tentative classifications for variants, which can be assessed during final case review. Systems that integrate such information into an internal variant database allow for progressive gains in analysis efficiency when the same variants are identified in future cases. A final consideration for selection of variant analysis platforms is support for downstream data sharing, which ultimately improves the quality of variant analysis. A list of platforms meeting minimum standards to support data sharing is available from ClinGen: https://clinicalgenome.org/tools/genomic-analysis-software-platform-list/.

Compound heterozygous SNV and CNV calls may not be readily identifiable by the interpretive tool in use. In this case, if a high quality CNV is detected containing genes with a known association to an autosomal recessive disorder and there is sufficient phenotypic overlap with the clinical presentation of the proband, re-examination of SNV data is recommended. Conversely, exon or gene-level CNVs and SVs may be specifically interrogated if a single heterozygous or apparent homozygous SNV is identified in a gene with autosomal recessive inheritance and sufficient phenotype overlap with the patient.

Variant prioritization can be considered as a means to improve overall analytical efficiency. By bringing the “low hanging fruit” (i.e., variants impacting genes that are highly relevant to the patient phenotype) to the top of the triage queue, downstream processes such as in-depth variant review and orthogonal confirmation can be completed while case analysis is ongoing. In medically urgent cases, prioritization is also used to quickly identify high-impact variants for preliminary results reporting. However, it is important to note that the complete set of variants meeting the genotype- and phenotype-centric filtering criteria described above should be reviewed for every case, even when a likely explanatory variant is identified early in the triage process. This is due to the possibility of identifying additional modifying factors or multiple genetic diseases in a single individual^74^. Prioritization therefore should not be expected to reduce the amount of time required to complete triage.

When evaluating the relevance of a variant to the patient phenotype, it is important to consider the possibilities of phenotypic expansion, variable expressivity, age-related penetrance, or additional syndromic features of a disease that may not have been recognized in the tested patient. This process is complicated by the fact that phenotypic feature prevalences are not well defined for most diseases, although this information is available for a subset of diseases in reference databases like DECIPHER (https://www.deciphergenomics.org/). While annotation resources are updated regularly, there may be a time-delay between a publication associating a variant or gene with a particular phenotype and an updated summary published in a database such as OMIM. As such, retrieving additional information from the most recent primary literature or other databases such as ClinVar may help the analyst accurately review variants identified via a genotype-driven approach. For interrogation of novel disease genes, candidate variants can be submitted to Matchmaker Exchange^75^ to determine if the gene has been implicated in a similar phenotype by other clinical or research laboratories. This approach has successfully provided diagnoses for many patients with rare disease^76^. However, reporting should not be delayed while waiting for Matchmaker Exchange results.

### Copy number variant analysis

Intragenic CNVs are typically triaged with the same considerations as SNVs or indels within the same gene; however, multigenic CNVs require additional considerations, both technical and interpretive. A preliminary step in CNV analysis should be a manual review of variant read depth and B-allele plots to ascertain the quality and accuracy of CNV calls^52^. Use of multiple SV callers, merging calls, and adjusting breakpoints may be required to ensure the accuracy of a CNV of interest given that callers often break large CNVs into multiple sequential, non-overlapping calls. In addition to the standard analysis of all CNVs affecting known disease-relevant genes or microdeletion sites, regardless of size, all multigenic CNVs meeting a certain size (typically > 500 kb for deletions and >1 Mb for duplications) or gene content threshold (e.g. more than 25 deleted genes), should be curated and classified per published guidelines^77^, and reported according to laboratory policy.

### In-depth variant assessment

When variants of interest are identified during triage, in-depth gene and variant analysis should be performed prior to making reporting decisions. Such analyses should follow existing guidelines on the interpretation of sequence variants^78,79^, copy number variants^77^, low penetrance/risk alleles (https://www.clinicalgenome.org/working-groups/low-penetrance-risk-allele-working-group/), mitochondrial variants^58,80,81^, and evaluating gene-disease relationships^82^.

### Secondary review

Given the large amount of information processed by analysts during triage steps, it is recommended that all triage decisions be agreed upon by at least two trained staff members. This QC step increases the accuracy of genome interpretations and aims to identify potentially missed candidate variants. Dual review increases the time and cost of the test, but the laboratory may efficiently design the review step in consideration of the type of personnel completing each step and the depth of review required to achieve high quality genome interpretation.

### Orthogonal confirmation or characterization of reportable variants

The decision to pursue orthogonal testing depends on the extent of the laboratory’s validation for a particular variant type, quality metrics for a specific variant and requirements from regulatory bodies^83–85^. For example, a laboratory may conduct orthogonal testing on the same specimen to confirm a variant with low quality metrics, confirm the result with a new specimen, clarify the exact repeat size for a locus with an expanded STR call using a targeted assay, deeply sequence (via a separate NGS test) an SNV to investigate potential of mosaicism, or determine levels of heteroplasmy in additional tissues from the affected individual.

## Reporting

Report contents should conform to existing guidelines for reporting from the ACMG^49^. Additional considerations particularly relevant to WGS reporting are presented below, including variants and genes of uncertain significance, STRs, and variants unrelated to the primary indication for testing.

Variants identified in established disease genes (as defined by current standards^82^) that are relevant to the primary indication for testing should be the primary focus of diagnostic WGS reports. In addition to those variants conferring a molecular diagnosis (e.g. heterozygous pathogenic (P) or likely pathogenic (LP) variants in dominant genes or biallelic P or LP variants in recessive genes), single heterozygous P or LP variants in a recessive disease gene that is highly specific for the patient phenotype should also be reported given the possibility of a missed “second hit”. For example, a variant in a low coverage region, a noncoding variant whose impact on gene expression has not been recognized, or a variant type outside of the laboratory’s validated test definition may all be missed by the test.

However, the complexity of genetic disease and magnitude of genetic information returned by WGS means that laboratories will also have to make reporting decisions for variants whose relevance to the patient is more ambiguous. Pertinent examples and overall approaches used by MGI laboratories are discussed in more depth in the Supplementary Discussion. Following these approaches, most participating laboratories report a typical range of 0–1 VUSs per patient (Supplementary Data 6).

WGS reporting policies should maximize the test’s diagnostic potential while minimizing the number of variants that may cause unnecessary clinician follow-up or patient stress or anxiety. Policies should be available to patients and ordering providers to ensure the patient is appropriately consented prior to the initiation of testing. Finally, laboratories are strongly encouraged to engage ordering providers in reporting decisions when there is uncertainty as to whether a variant aligns with the patient’s phenotype or the family’s preferences. This communication and the factors considered in the decision to return a result should be documented in the laboratory’s record.

### Result summary

While it is common practice for targeted panel results to be classified as “Positive”, “Negative”, or “Inconclusive” based on the variants identified, the definition of these categories becomes more complex in genomic testing, which may identify variants relevant to the primary indication for testing, variants that explain non-primary phenotypes, secondary findings, or other variant types. We support current ACMG recommendations^49^, which state that “Primary findings in a diagnostic test should appear as a succinct interpretive result at the beginning of the report indicating the presence or absence of variants consistent with the phenotype.” Laboratories may find terms such as “Positive” or “Negative” useful for straightforward WGS results, but a descriptive statement defining those terms (e.g. “Positive: Findings explain indication for testing”) should also be considered. When the interpretive result is more complex, descriptive statements that speak to the relevance of the result to the patient phenotype are essential. The integration of WGS results into the medical record should be considered when drafting interpretive result summaries, since the summary may influence whether or not a provider chooses to review the report in detail. It is also possible that future clinical informatics standards (e.g. HL7) may dictate that each indication for testing has a separate and distinct result. Therefore, considering report structure in light of these evolving standards may be prudent.

### Technical limitations

Reporting practices for technical limitations should be consistent with other NGS-based testing standards^49^. In addition to listing key components of the bioinformatics pipeline, the description of the analysis strategy should define all filtering and/or prioritization approaches used. The known limitations of the testing methodology as well as any variant types not interrogated should be described. Any known technically challenging regions or coverage issues in genes that are likely to be highly relevant to the patient (e.g. limited sensitivity for F8 inversions in a patient with hemophilia^86^) should be specifically called out as a potential source of reduced sensitivity. Several tools for calculating region-specific coverage are freely available^87–89^.

## Reanalysis

Periodic case reanalysis has been demonstrated to improve diagnostic yield^90–93^. It is therefore recommended that laboratories provide an option for reanalysis of finalized WGS cases. Ideally, reanalysis policies should be developed in advance of test launch and communicated to providers at the time the test is ordered. The ACMG has recently produced two “points to consider” documents relevant to reanalysis policies and procedures^94,95^. Of note, procedures for variant-level reevaluation are not substantially different from other genetic testing methods and have been addressed elsewhere^78,94^.

Robust reanalysis may necessitate re-running multiple steps of the test (Table 2). Given the significant role that the patient phenotype plays in the analysis process, laboratories should request updates to the patient’s medical and family history prior to initiating reanalysis. Phenotype- and genotype-driven analyses should be reviewed and adjusted in light of the updated patient information.

**Table 2:** Suggested steps of reanalysis based on events that have occurred since initial analysis.

			Tertiary analysis			
Change since initial analysis	Primary analysis (sample/library prep and sequencing)	Secondary analysis (mapping, alignment, variant calling, QC)	Annotation	Variant stratification	Variant and gene assessment	Reporting
Significant improvements in library prep/sequencing technology	✔	✔	✔	✔	✔	✔
Bioinformatics improvements		✔	✔	✔	✔	✔
>1 year lapsed since initial analysis			✔	✔	✔	✔
Additional patient phenotypes or family history				✔	✔	✔
Improved understanding of the genetic etiology of patient condition				✔	✔	✔
New methodology or resource for variant assessment					✔	✔

Even in the absence of new phenotype information, newly published variant or gene-level evidence may allow a previously unreviewed variant to meet criteria for expert review or a previously reviewed variant to meet criteria for reporting. Laboratories may consider suggesting a minimum time period (e.g. one year) to have elapsed since the initial analysis to conduct this type of case review. Alternatively, reanalysis may be initiated by new publications, updated transcripts and new gene models, or changes to the WGS test definition that may impact a set of cases.

The initiation of reanalysis may be either reactive (i.e., when requested by the ordering provider/patient) or proactive (independently triggered by the lab). As of the time of this publication, the majority of laboratories currently offering WGS analysis are performing reactive reanalysis. However, proactive reanalysis, including the acquisition of updated clinical information, is recognized as an important step towards maximizing the clinical utility of the WGS test. Several key issues stand in the way of this ideal, including the personnel effort required to conduct analysis, lack of reimbursement, limited tools to enable tracking of cases in need of follow-up, and limited tools to automatically identify new scientific literature relevant to variants and genes reviewed in past cases. Improved systems to address these issues will be critical to the future implementation of proactive reanalysis.

Given the substantial effort required for case reanalysis, laboratories may need to charge a fee for this service. Furthermore, given the differing approaches, algorithms, and professional opinion inherent to WGS analysis, laboratories should support the sharing of raw sequencing data and other file types to enable analysis by other laboratories or research programs if requested by the patient or ordering provider. MGI laboratories are currently providing this data through encrypted hard drives or access through a portal.

## Key recommendations

Key recommendations are summarized below. Given our goal to provide a complete reference for WGS, this list includes recommendations that are also relevant to WES.

Requisition/Consent

- While the use of structured phenotype ontologies is important for the automation and scalability of WGS, laboratories are cautioned against requiring ordering providers to submit information in the primary structured form given the potential for loss of detailed and nuanced phenotypic information
- The test ordering process should enable physicians to specify the primary clinical question of interest
- Test requisition forms and any laboratory provided consent forms should clearly indicate that genetic variants relevant to any phenotype provided to the laboratory may be returned unless specific reporting instructions are provided by the ordering clinician
- For trio or other multiple-family member sequencing approaches, requisition and/or consent forms should clarify how the data from auxiliary family members will be used and reported
- Phenotypic data submitted with the test order should undergo review by laboratory staff prior to the initiation of testing and laboratories should seek clarification of unclear or conflicting information
- Policies for secondary findings analysis and secondary and incidental findings reporting should be developed in advance of launching a WGS test and clearly defined on the laboratory’s requisition and any provided consent form

Annotation

- In the absence of infrastructure that can support continuous updates, we recommend that data sources used in annotation pipelines be updated at least quarterly.
- Until noncoding regions of the genome can be systematically interrogated and interpreted, laboratories should ensure that their pipelines are able to capture all known pathogenic variants in ClinVar, including those that fall outside of coding sequence and flanking intronic regions.

Analysis

- The overall analysis approach should incorporate both genotype-driven and phenotype-driven strategies.
- The complete set of variants meeting the genotype- and phenotype-centric filtering criteria should be reviewed for every case, even when a likely explanatory variant is identified early in the triage process.
- If the interpretive tool in use does not readily identify compound heterozygous calls across variant classes (e.g. SNV and CNV), re-examination of calls across all relevant variant classes should be performed when a compelling monoallelic variant is found in an autosomal recessive gene associated with the patient’s phenotype.
- Given the large amount of information processed by analysts during triage steps, it is recommended that all variants identified by genotype- and phenotype-centric filtering criteria be reviewed by at least two trained staff members.
- The sensitivity and specificity of automated analysis tools remain insufficient to replace the role of a human analyst. Results from automated analyses should be carefully assessed by a human analyst prior to consideration for reporting.

Reporting

- The laboratory should establish policies defining the types of findings that are considered for return, and these policies should be available to patients and providers.
  - Variant-level evidence, gene-level evidence, and the correlation between the patient’s phenotype and the gene should be addressed.
  - The goal of reporting policies should be to maximize the test’s diagnostic potential while minimizing the number of VUS reported.
- We strongly encourage open communication between ordering providers and the laboratory regarding reporting decisions, particularly for more challenging cases (e.g. if there is uncertainty as to whether a finding aligns with patient phenotype).
- When summarizing WGS findings on reports, laboratories may find terms such as “Positive” or “Negative” useful for straightforward results, but a descriptive statement defining those terms (i.e. “Positive: Findings explain indication for testing”) should also be considered. When the interpretive result is more complex, high-level descriptive statements that speak to the relevance of the result to the patient phenotype are essential.

Reanalysis

- It is highly recommended that laboratories provide an option for reanalysis of WGS cases. A charge for this service is acceptable.

## Key unmet needs

Requisition/Consent

- Phenotype capture methods maximize the amount of patient and family history information available to laboratories in a structured, machine-readable format without placing unnecessary burden on clinicians.

Annotation

- Standardization of NGS data annotations.
- Annotations to support analysis of a broader range of molecular pathogenic mechanisms (e.g. in silico predictors for noncoding variants that affect promoters or coding variants that affect exonic splice enhancers, and databases cataloguing structural variation within large populations).

Analysis

- Routine deposition of variant data in structured format into centralized databases (e.g. ClinVar) and scientific literature to improve identification of previously reported variants.
- Improved tools for calling, filtering, and interpretation of SVs and STRs.
- Maturation and validation of AI/machine learning tools will be needed to scale analysis.

Reporting

- Structured integration of WGS results into the medical record or connected platforms.

Reanalysis

- Tools to support systematic proactive reanalysis (i.e., tools to automatically identify new scientific literature relevant to variants and genes reviewed in past cases).
- Reimbursement for reanalysis.

### Reporting summary

Further information on research design is available in the Nature Research Reporting Summary linked to this article.

## Supplementary information

Supplementary Information

Supplementary Data 1

Supplementary Data 2

Supplementary Data 3

Supplementary Data 4

Supplementary Data 5

Supplementary Data 6

Reporting Summary Checklist

## Data Availability

All structured data generated or analyzed during this study are included in this published article (and its supplementary information files).
