# Supplementary material for: Best practices for the interpretation and reporting of clinical whole genome sequencing
Source: NPJ Genom Med. 2022 Apr 8;7:27. doi: 10.1038/s41525-022-00295-z (PMC8993917; doi:10.1038/s41525-022-00295-z)
Supplement: Supplementary file 1 — Supplementary Information [file 41525_2022_295_MOESM1_ESM.pdf]

Supplementary information: CA Austin-Tse et al., *Best practices for the interpretation and reporting of clinical whole genome sequencing*

Table of contents

Index of Supplementary Data Available in Separate Files 2

Medical Genome Initiative Members 3

Supplementary Note 1: Informal polling questions 4

Supplementary Note 2: Sample WGS requisition form 19

Supplementary Note 3: Essential elements of WGS consent 25

Supplementary Discussion: Additional analysis, prioritization, classification, and reporting considerations..31

Supplementary Figure 1: Personnel involved in WGS tertiary analysis 34

Supplementary References...35

Index of Supplementary Data Available in Separate Files

**Supplementary Data 1**	Data annotation sources utilized in the WGS pipelines of participating MGI laboratories
**Supplementary Data 2**	Example calling methods for clinically relevant variants detectable by WGS
**Supplementary Data 3**	Resources used by participating institutions in WGS triage steps, including variant and gene-disease association databases, allele frequency databases, RNA and protein expression data, and visualization tools
**Supplementary Data 4**	Examples of low penetrance, risk, and other variants of interest that may be excluded from filtering results due to high frequency in population databases
**Supplementary Data 5**	Sources of gene-disease association information used for gene list curation by participating MGI laboratories
**Supplementary Data 6**	Case analysis statistics from participating MGI laboratories, including number of variants triaged per case, average case analysis time, and number of VUS per report

Medical Genome Initiative Members

Euan Ashley[15], David Dimmock[12], James (Matthew) Holt[7], Vaidehi Jobanputra[4,5], Hutton M Kearney[10], Shashikant Kulkarni[13,14], Christian Marshall[9], Heidi L Rehm[1,3], Ryan J Taft[6]

Supplementary Note 1

This document contains an outline of the informal polling questions used by the Medical Genome Initiative WGS interpretation and reporting working group to gather information about practices in participating laboratories. Responses were discussed via teleconference and used to guide our recommendations. Key results are summarized in the manuscript and supplementary materials.

Requisition/Case Demographics:

1. How does your lab collect patient clinical information when a WGS test is ordered? Select all that apply
    a. Open text field for clinician to write down clinical presentation
    b. Series of targeted questions to collect comprehensive clinical data in written form
    c. Laboratory collects clinical information over the phone
    d. Referring physician provides medical records
    e. Laboratory has access to the electronic medical record
    f. Electronic tool is used by ordering clinician to supply clinical presentation (PhenoTips, PhenoDB, Face2Gene, custom tool, etc.)
    g. Other – please explain

2. How often do you receive clinical information in the form of HPO terms?
    a. Never
    b. Rarely
    c. ~ 50% of cases
    d. Most of the time
    e. Always

3. Indicate the proportion of WGS cases at your lab for which you receive the following types of clinical information.

	Never	Rarely	Sometimes	Most of the time	Always
Suspected clinical diagnosis					
One or more phenotypes relevant to suspected diagnosis/primary indication for testing					
Additional phenotypes that may or may not be relevant to the primary					

indication for testing					
Clinic notes					
Full medical record					
Family history					
Results from previous genetic testing					
Other (please describe)					

4. Describe the lab's process for reviewing the phenotype and clinical history of the individual being tested. Please note if there is a minimum amount of clinical information required to accept a case for WGS analysis.

5. Indicate the proportion of diagnostic WGS cases tested at your lab that match the family structures listed below. Please use the comments section to indicate any specific family structure requirements for your WGS test.

	0-10% of cases	10-40% of cases	40-60% of cases	60-90% of cases	>90% of cases	Not accepted
Singleton/proband only						
Trio (Proband and Parents)						
Multiple affected family members						
Other (please describe)						

6. Indicate the approximate proportion of cases tested at your lab where the proband is in the following age groups

	0-10% of cases	10-40% of cases	40-60% of cases	60-90% of cases	>90% of cases	Not accepted

Prenatal						
Neonatal/infant						
Pediatric						
Adult						

Annotation/Filtration - **Please provide a list of variant annotations used in your genome analysis pipeline** in addition to answering the questions below:

7. What program(s) do you use for WGS annotation? Select all that apply, and provide details on the program(s) used in the comments.
   a. Open source
   b. In-house proprietary build
   c. Commercially available filtration system
   d. Combination of commercial/open source/proprietary software

8. What program(s) do you use for WGS filtration? Select all that apply, and provide details on the program(s) used in the comments.
   a. Open source
   b. In-house proprietary build
   c. Commercially available filtration system
   d. Combination of commercial/open source/proprietary software

9. In addition to SNVs/small indels, which of the following variant types is called by your WGS pipeline? Select all that apply. If any of these variant types are called in a subset of diagnostic genomes run at your lab, use the comments to elaborate on the situations in which they are called. Please also indicate if annotation and filtration programs other than those described in questions 7 and 8 are used for these variant types.
   a. CNVs (del/dup)
   b. Other SVs (inversion/translocation/complex rearrangements)
   c. Mitochondrial variants
   d. Known repeat expansions
   e. Novel repeat expansions
   f. Mosaic variants
   g. Regulatory elements

10. Does the filtration program used in your lab incorporate automatic prioritization of variants based on patient phenotype?
    a. No, filtration is used to limit the number of variants analyzed, but variants are NOT prioritized based on phenotype

    b. Yes, filtration is used to limit the number of variants analyzed AND variants returned by the filtration are prioritized based on phenotype
    c. Yes, variants are prioritized based on phenotype without a filtration step
    d. Other (please describe)

11. Is AI incorporated into your variant filtration/prioritization methods? If yes, please describe how AI is used.

12. If possible, provide a summary of all data filtration strategies used in your lab (including the purpose and methodology behind each filtration). Please consider all variant types and family structures in your response.

13. Does your lab incorporate a phenotype-agnostic filtration in WGS analysis?
    a. No, we do not incorporate phenotype-agnostic filtration
    b. Yes, phenotype-agnostic filtrations are applied to all cases
    c. Yes, phenotype-agnostic filtrations are applied to a subset of cases (please specify)

14. If not already addressed in question 12, how is the phenotype-agnostic filtration structured?

15. Does your lab incorporate a phenotype-driven/gene list-based filtration in WGS analysis
    a. No, phenotype is used for variant prioritization/triage but NOT variant filtration
    b. Yes, phenotype-driven filtrations are applied to all cases
    c. Yes, phenotype-driven filtrations are applied to a subset of cases (please specify)

16. If not already addressed in question 12, how are the phenotype-driven filtrations structured?

17. Are phenotype-specific virtual gene panels (gene lists) used in any part of the WGS analysis process? If yes, please describe how gene lists are used.

18. If gene lists are used, where do they come from?
    a. Gene lists are manually curated
    b. Gene lists are automatically generated by a program (please specify in comments)
    c. Pre-made gene lists from an external source are used (please specify source in comments)
    d. Other (please specify)

19. If gene lists are manually curated, what resources are used during curation (select all that apply)?
    a. ClinVar
    b. Gene Atlas
    c. Genetic Association Database
    d. GeneReviews

e. HGMD Professional Version
f. HuGE Navigator
g. Human Phenotype Ontology
h. KEGG Disease Database
i. OMIM
j. Phenomizer
k. PubMed
l. SimulConsult
m. DisGeNet
n. PanelApp
o. Other (Please describe)

20. Are genes of uncertain significance (GUS) included on the gene lists you use for variant filtration?

21. How often are gene lists reviewed/updated?
    a. New gene lists assembled for every case
    b. No specific policy
    c. Weekly
    d. Monthly
    e. Quarterly
    f. Semi-annually
    g. Annually
    h. Other (please specify)

22. For patients with multiple distinct phenotypes, do you create a separate gene list/filtration for each phenotype?

23. Describe any QC processes in place to verify that gene lists are sufficiently comprehensive. Who performs this task (Genetic Counselor/Lab Director/Variant Scientist/etc.)?

24. Does your lab incorporate an inheritance-pattern driven approach in WGS filtrations (ie rare biallelic variants in recessive genes)
    a. No, we do not incorporate inheritance-pattern driven filtrations
    b. Yes, inheritance-pattern driven filtrations are applied to all cases
    c. Yes, inheritance-pattern driven filtrations are applied to a subset of cases (please specify)

25. If not already addressed in question 12, how are inheritance-pattern driven filtrations structured?

26. Does your lab incorporate a separate filtration strategy to identify secondary findings?
    a. No, we do not offer secondary findings analysis

   b. No, we use the phenotype-agnostic filtration (or another existing filtration - please specify) to identify variants relevant to the primary indication AND secondary findings
   c. Yes, we run a specific filtration for secondary findings

27. If you do run a specific filtration for secondary findings, how is that filtration structured? (Skip if already addressed in question 12)

28. Which genes are considered for secondary findings analysis and reporting (ie active screening for variants unrelated to the primary indication for testing)?
    a. We do not offer secondary findings analysis
    b. ACMG recommended list only (currently 59 genes)
    c. ACMG list + additional selected genes (please describe)
    d. Custom list that does not overlap with- or only partially overlaps with- the ACMG recommended genes (please describe)
    e. Any gene in which a clearly disease-causing variant is found
    f. Other (please specify)

29. Do you have a unique policy for analysis and reporting of <u>incidental</u> findings (ie variants unrelated to the primary indication for testing that were incidentally discovered via other analysis methods)? If so, please describe.

30. Please summarize any WGS filtration methods routinely utilized in your lab that are not captured in the questions above

31. Please indicate if there is a preferred order to the analysis strategies performed for a typical WGS case in your lab (i.e. phenotype agnostic first, then phenotype-specific).

<u>Triage</u> (For the purposes of this poll, triage includes the review of variants returned by the filtration methods above and decision making regarding which variants will be included on the final report)

32. Are all variants returned by the filtration methods used in a typical WGS case triaged? If no, please estimate the proportion of variants that are excluded from triage and describe how variants are selected for triage:

33. How many SNVs/small indels are triaged in a typical WGS case (across all filtration methods used)?

	0-10	11-50	50-100	100-200	>200
Singleton					
Trio					

34. How many total CNV/SVs are triaged in a typical WGS case (across all filtration methods used)?

	0-10	11-50	50-100	100-200	>200
Singleton					
Trio					

35. If other variant types (outside of SNVs/small indels and CNVs/SVs) are routinely analyzed, please describe the variant types and the number of variants typically triaged per case.

36. How many stages of triage are typical for a WGS case in your lab prior to final review by a lab director?
    a. Primary review only
    b. Primary review and secondary review
    c. Two primary reviews conducted in parallel
    d. Other (please describe)

The following questions pertain to your lab's process for triaging variants:

37. Do all variants undergo full ACMG-style assessment? A modified, rapid assessment? Combination of both? Please provide a description of your variant assessment process and estimate the proportion of variants that undergo each assessment style.

38. Are all variants classified (B/LB/VUS/LP/P) during triage? If no, please describe which variants are classified.

39. Does your lab utilize VUS subcategories (VUS-favor pathogenic/VUS/VUS-favor benign) when classifying variants?

40. When evaluating gene/disease associations how often are each of the following sources used? If you routinely use a source for evaluating gene/disease associations that is not listed in the table below, please specify.

	Not used	Occasionally consulted	Often consulted	Usually consulted	Always consulted
ClinVar					
Genetics Home Reference					
GeneReviews					
HGMD					
OMIM					
PubMed					
DisGeNET					
Google					

41. When considering the reportability of a variant, how often are each of the following resources used?

	Not used	Occasionally consulted	Often consulted	Usually consulted	Always consulted
ClinVar					
Genetics Home Reference					
GeneReviews					
HGMD					
OMIM					
PubMed					

DisGeNET					
Google					
In silico prediction tools (SIFT/Polyphen/etc.)					
gnomAD/ExAC					
GTEx					

42. Does your lab consider variants in genes of uncertain significance for inclusion on WGS reports? If so, please describe the situations in which these variants might be returned (or if they are routinely returned)?

43. How do you decide when case analysis is complete? (For example, do you stop triage once you find an explanation for the patient phenotype? Do you have a minimum set of filtrations that must be triaged regardless of whether an explanation is found?)

44. Other comments on how variant triage is performed?

<u>Reporting</u> - **If possible, please provide a sample report** in addition to answering the questions below:

45. Which variants are reported for diagnostic WGS (select all that apply)?
    a. Relevant to primary phenotype
        i. Pathogenic
        ii. Likely pathogenic
        iii. Uncertain significance
        iv. Likely benign
        v. Benign
    b. Relevant to additional phenotypes (not the primary indication for testing)
        i. No variants relevant to additional phenotypes reported
        ii. Pathogenic
        iii. Likely pathogenic
        iv. Uncertain significance
        v. Likely benign
        vi. Benign
    c. Secondary/incidental findings
        i. No secondary findings reported
        ii. Pathogenic
        iii. Likely pathogenic
        iv. Uncertain significance

v. Likely benign
vi. Benign

d. Risk
    i. Risk variants not reported
    ii. Defined list of risk alleles is assessed regardless of phenotype
    iii. Risk would only be reported if relevant to patient phenotype
    iv. Other (please specify in comments)

e. Pharmacogenomics (PGx)
    i. PGx variants not reported
    ii. Defined list of PGx alleles is assessed regardless of phenotype
    iii. PGx would only be reported if relevant to patient phenotype
    iv. Other (please specify in comments)

46. Describe your lab's confirmation methods for reported variants (select all that apply)
    a. All reported sequence variants are confirmed by orthogonal methods (both diagnostic and incidental findings)
    b. Only diagnostic findings are confirmed by orthogonal methods
    c. No variants are confirmed by orthogonal methods
    d. Other (please explain)

47. Are there situations in which your lab would relay results to the clinical provider prior to variant confirmation or the distribution of a finalized report? If yes, please provide details on the situations in which this would be done and how results are communicated.

48. Do your genome reports include an overall interpretation of the results (i.e. positive/negative/inconclusive or solved/unsolved)? If yes, please indicate categories used and the types of results that fit into each category.

49. Indicate whether your genome reports contain the following information, and if the information is present on the main report or a report supplement. If a piece of information would only be reported in specific situations, please use the comments column to describe when they would be reported.

	Not included on reports	Main report	Report supplement	Comments
Description of WGS analysis approach				
High-level summary of results				
Limitations of the WGS analysis				
Variants clearly				

relevant to primary indication				
Variants with possible (but unclear) significance to primary indication				
Variants clearly relevant to additional phenotypes (not primary indication)				
Secondary/incidental findings variants				
Risk alleles				
PGx variants				
Coverage metrics for genes of relevance to patient phenotype				
Carrier status variants relevant to patient phenotype				
Carrier status variants NOT relevant to patient phenotype				

50. Are SNVs/small indels reported in genes of uncertain significance (GUS)?
    a. No, we do not report variants in GUS's
    b. Yes, if the gene has been associated with a phenotype that is a good match for the patient disease
    c. Yes, only when the gene has been associated with a phenotype that is a good match for the patient disease AND the variant occurred de novo
    d. Other (please specify)

51. Are other variant types reported in genes of uncertain significance? If yes, please provide details.

52. What follow-up is recommended for unsolved cases?
    a. None
    b. Reanalysis (please indicate suggested timeframe if any)
    c. Referral to research projects

    d. Referral to Matchmaker Exchange or similar databases
    e. Follow-up testing (please indicate types of testing recommended)
    f. Other (please specify)

53. Do you routinely share raw data with patients/ordering providers?
    a. Raw data is automatically shared for every case
    b. Raw data is automatically shared for a subset of cases (please specify)
    c. Raw data is shared only if specifically requested
    d. We do not share raw data

54. If you do share raw data, is there a fee for that service?

55. What forms of raw data will you share if requested (select all that apply)
    a. Fastq
    b. Bams
    c. Vcf
    d. Annotated vcf
    e. Variant filtration/triage outputs
    f. Other (please specify)

56. If your lab operates within a medical center, is raw genome data associated with the patient medical record?

<u>Variant reclassification</u>

57. Does your lab ever submit variants identified in genome analyses to Matchmaker Exchange?
    a. Yes
    b. No

58. If variants are submitted to Matchmaker Exchange, how do you manage these submissions and the resulting follow-up?

59. Please describe your lab's variant reclassification policy: When is variant reanalysis performed? How are changes in classification reported to the ordering provider?

<u>Reanalysis</u> - For the following questions, re-analysis is defined as a process that, at minimum, involves a new round of variant triage. Requests for reassessment of single variants are NOT considered re-analysis.

60. Does your lab offer re-analysis of finalized WGS cases?
    a. No (skip to next section)
    b. Yes (answer questions below)
61. Select the situations your lab would accept as a valid indication for reanalysis (select all that apply). If additional stipulations apply to any of these indications, please elaborate in comments.

a. New patient phenotype recognized
b. Genes relevant to primary indication missed in initial analysis
c. Genome data available for additional family members
d. No new patient information, but it has been ____months/years since initial analysis (provide minimal acceptable time period if applicable)
e. Change to lab informatics pipeline
f. Other (please specify)

62. How is reanalysis initiated? (i.e. reanalysis of unsolved cases is automatically triggered after a certain time period, or reanalysis is only performed on request)

63. What is the fee structure for reanalysis? Select all that apply
    a. No specific policy defined
    b. All reanalyses performed free of charge
    c. First reanalysis is free, additional reanalyses incur a charge
    d. Reanalysis fee (if any) depends on the indication for reanalysis or the extent of reanalysis requested (provide details in comments)
    e. All reanalyses incur a fee
    f. Other (please specify)

64. Describe filtration/analysis approaches that would be used for the following indications for reanalysis.
    a. New patient phenotype recognized
    b. Genes relevant to primary indication missed in initial analysis
    c. Genome data available for additional family members
    d. No new patient information, but acceptable minimum time period has elapsed since initial analysis
    e. Change to lab informatics pipeline
    f. Other (please specify)

65. When would you recommend resequencing vs. reanalysis?

Personnel/Effort

66. Indicate who typically performs each step of the WGS analysis process

	PhD-level ABMGG fellow/MGP resident in training	PhD-level trained analyst, not board certified and not in an ABMG/MGP training program	Genetic counselor	BA/MS level staff	Board-certified clinical laboratory geneticist	Board-certified molecular genetic pathologist	Other (please describe)	N/A

Extraction of key phenotypes from submitted clinical information								
Gene list curation								
Triage								
Secondary review								
Report drafting								
Final case review								

67. How many individuals are directly involved in the interpretation of a typical genome case at your lab (not counting ordering providers)?
    a. 1
    b. 2
    c. 3
    d. 4 or more
68. Indicate the average total time spent on interpretation of a typical case, including all individuals directly involved
    a. <2 hrs.
    b. 2-4 hrs.
    c. 4-8 hrs.
    d. 8-10 hrs.
    e. 10-12 hrs.
    f. 12-14 hrs.
    g. >14 hrs.
69. Please indicate how often (if at all) ordering providers might be consulted during the following stages of the WGS analysis process

	Never	Rarely	Occasionally	Frequently	The majority of cases

Clarification of patient phenotype prior to analysis					
When classifying a variant					
When debating relevance of a variant to the primary indication for testing					
When debating relevance of a variant to additional phenotypes (outside of primary indication)					
When debating reportability of a secondary/incidental findings variant					

Other

70. What do you consider to be the most significant pain points in the WGS analysis process?

Supplementary Note 2

A sample clinical WGS requisition form is provided on the following page. **This sample form is provided for informational purposes only and does not represent requisition forms in use at MGI participating institutions.**

Patient Name:
MRN:
DOB:

Genome Laboratory
23 DNA Way
Mendel, CT 31415

SAMPLE Whole Genome Sequencing Test Requisition

DISCLAIMER: This sample form is provided for informational purposes only and does not represent requisition forms in use at MGI participating institutions.

Patient Information

First name: ____________________________ MI:____ Last name: __________________________________
Date of Birth: (mm/dd/yyyy) ______ /______ / ________ Gender: ❑ Male ❑ Female ❑ Unknown/Unspecified
Address:____
City: ___________ State: _____ Zip Code: __________ Phone:_______________________________________
Email: ____
Institution:____________________________________ Medical Record Number: _______________________
Is the patient adopted? ❑ Yes ❑ No Is the patient deceased? ❑ Yes ❑ No *if yes,* date:_____

Has the patient undergone bone marrow transplant? ❑ Yes ❑ No *if yes,* date: _______________
Testing for patients who have received an allogenic bone marrow transplant must be completed on a pre-transplant sample or a non-hematologic sample.

Has the patient received a blood transfusion? ❑ Yes ❑ No *if yes,* date: _______________
Blood obtained for genetic testing should ideally be collected at least 2-4 weeks after the date of the last transfusion

Race and Ethnicity (please select all that apply):
❑ White ❑ Ashkenazi Jewish ❑ Hispanic ❑ Asian ❑ Black/African American ❑ American Indian/Native Alaskan
❑ Native Hawaiian or other Pacific Islander ❑ Other

Provider Information

Referring Provider
Name: _______________________________________ Phone: ____
Department:___________________________________ Fax:____
Institution: ___________________________________ Email: ____
Address: ____ City: ______________________________________
State:____________________________ Zip Code: _______________ Country: ________________________________

Genetic Counselor/Additional Contacts
Name: _______________________________________ Phone: ____
Fax: ____ Email: ____
Institution/Address: ❑ Same as Referring Provider ❑ Provided below
____
____

Who should the laboratory contact with questions about this case? ____

Patient Name:
MRN:
DOB:

Genome Laboratory
23 DNA Way
Mendel, CT 31415

SAMPLE Whole Genome Sequencing Test Requisition

Sample Information

❏ **Blood** (5-7mL K_2EDTA/K_3EDTA)
Date Collected: (mm/dd/yyyy) ______ / _____ / _______

❏ DNA - Source: _________________________

❏ Other ________________________________

Test Order

❏Proband Only Clinical Genome Sequencing + Interpretation.
❏Trio (or other family-based analysis) Clinical Genome Sequencing + Interpretation.

For family-based analysis, submit a test requisition form for each patient submitted for testing.
Number of family members submitted for testing __________________

	Affected Status		
If axillary family member, relationship to the proband:	Affected	Unaffected	Unknown
❏ Biological mother of the proband	❏	❏	❏
❏ Biological father of the proband	❏	❏	❏
❏ Full brother of the proband	❏	❏	❏
❏ Full sister of the proband	❏	❏	❏
❏ Other [describe relationship to the proband specifically (eg, maternal half-sister of the proband)]	❏	❏	❏

❏Reanalysis/Reinterpretation of Clinical Sequencing Data
- ❏Reanalysis of whole genome
- ❏Reanalysis of selected genes of interest (provide details below or contact the laboratory)
  Genes requested: ____

Genome Laboratory
23 DNA Way
Mendel, CT 31415

SAMPLE Whole Genome Sequencing Test Requisition

Clinical Information

Clinical information is crucial for accurate interpretation of results. Please provide the primary indication for testing in addition to completing at least one of the three options for detailed phenotype submission below. Failure to provide clinical information will result in delay of testing

Primary indication for testing: ____

____

ICD-10 codes:____

The WGS report will contain any identified variants that are suspicious for causing the symptoms/diseases provided. If there is a specific condition that might cause your symptoms but you do NOT wish to learn about, please discuss it with your doctor and/or genetic counselor and list here:

____

____

Detailed phenotype information (select at least one option below):

❑Option 1: Include a recent clinic note, including family history; Photo(s) of the patient may be helpful if available.

❑Option 2: Submit clinical information via [phenotype interface of choice, e.g. PhenoTips]

❑Option 3: Complete section below

Abnormality of:

Head or neck	
Eye	
Ear	
Voice	
Thoracic cavity	
Cardiovascular system	
Breast	
Respiratory system	
Limbs	
Musculature	
Skeletal system	
Connective tissue	
Digestive system	

Genome Laboratory
23 DNA Way
Mendel, CT 31415

SAMPLE Whole Genome Sequencing Test Requisition

Nervous system	
Genitourinary system	
Immune system	
Endocrine system	
Blood and blood-forming tissues	
Metabolism/homeostasis	
Integument	
Growth abnormality	
Prenatal development or birth	
Neoplasm	
Other	

Secondary Findings

The primary goal of the WGS test is to find the genetic basis of your/your child's disorder. However, a secondary findings analysis is also available. This analysis includes a targeted screen for disease-causing variants in medically actionable genes that may be unrelated to your/your child's disorder but are recommended for reporting by the American College of Medical Genetics and Genomics (ACMG). This analysis is optional.

❑Patient **OPTS IN** to secondary findings analysis of the ACMG recommended genes

❑Patient **OPTS OUT** of secondary findings analysis

Important points to consider:
- Opting out of secondary findings analysis means that a targeted search for variants in the list of genes recommended by the ACMG for reporting of secondary findings will not be performed.
- If an individual opts out of the analysis, **variants in secondary findings genes may still be reported if they have possible relevance to the indication for testing**.
- In the case of a family-based analysis (e.g., trio sequencing), identification of secondary findings in family members who opt in for the analysis may inform carrier status of other members of the family, even those who choose to opt out of the analysis.

Genome Laboratory
23 DNA Way
Mendel, CT 31415

Incidental Findings

Incidental findings are defined as clinically significant variants found in genes associated with phenotypes that are unrelated to the patient's primary indication for testing. Unlike Secondary Findings, these variants are not actively sought, but may be noted during analysis. Variants in genes that are not part of the ACMG recommended gene list for Secondary Findings but have the potential to influence medical management [insert language for how these variants will be handled/reported by the laboratory per the laboratory's incidental findings policy].

Physician Signature

Please review the Patient Consent Form with your patient prior to ordering this test.

I certify that (i) the patient (or authorized representative on the patient's behalf) has given his/her informed consent (which includes written informed consent or written authorization when required by law) to have this genetic test performed, (ii) the informed consent obtained from the patient meets the requirements of applicable law and the Patient Consent Form, and (iii) I am a medical doctor with the proper licensing in my country to order this testing. I agree to provide [Laboratory], or its designee, any and all information reasonably required for this genetic testing to be performed.

Authorized Physician Signature (required) Date (MM/DD/YYYY)

Supplementary Note 3

Here we list key concepts that are recommended for inclusion in WGS consent. Sample text for each concept is provided. The sample text is based on consent language currently in use at participating laboratories. We provide these samples as an aid to WGS laboratories. The **text should be edited** as needed to fit the context of the test offering and according to institutional policy and the laws and cultural norms of the state/country/countries involved.

**Concept**	**Sample Text**
Definition of WGS	Genome testing examines many genes in your body to look for a genetic cause for your health issues. Our genes are the instructions in our cells that determine who we are. They influence things like whether we are tall or short or have blue or brown eyes. Sometimes they also influence whether we will develop a certain illness. This test looks for genetic changes that are associated with specific diseases.
Definition of testing process	You/your child will be asked to provide a sample for testing such as a blood sample or other tissue. Your provider will share your/your child's medical and family history information to the lab to help interpret the results of testing. The lab may also ask for samples from other family members to help do the test. The lab will generate the DNA sequence for your genome and look for differences in genes that are related to your medical condition. This test will **not** tell you about all the differences in your genes that may affect your health. If you have other health issues in your family besides the main reason you are having this test, please talk with your provider. Otherwise, they will **not** automatically be looked at.<br><br>A written report of your results will become part of your electronic medical record. Your doctors and potentially your health insurance providers will have access to this information. We will only issue a test report for the person on whom the test is being done. In that report, we may mention if the genetic differences are inherited from a parent. We will not provide reports for family members who give samples to help with the test analysis unless specifically requested. The healthcare provider who ordered the test will contact you with results. After testing is completed, further medical or family history information, or further medical tests may be suggested to help interpret the results of testing.

Risks of WGS testing	This test requires DNA, which is most often provided from a sample of blood. Side effects of having blood drawn are uncommon, but may include dizziness, fainting, soreness, bleeding, bruising, and rarely, infection.<br><br>Additionally, you may learn medical information about yourself or your family members that you did not expect as a result of this test. Examples include ancestry and risk/predisposition to a disease that may or may not be preventable or treatable. You may learn that family relationships are not what you thought. For example, sometimes we discover that someone's biological father is not who they thought. You or your family members may be upset to learn about the types of information described above.<br><br>Some people are concerned that genetic information could be used to discriminate against them. There is a federal law (called "GINA," the Genetic Information Nondiscrimination Act of 2008) and state laws to prevent employment and health insurance discrimination. However, there may not be laws to prevent the use of genetic information on the ability to obtain life, disability, or long-term care insurance. Talk to your doctor or a genetic counselor if you are worried about this.
Limitations of WGS testing	This test cannot sequence all parts of a person's genome and some types of genetic differences cannot yet be found with this test. No genetic test can look for all genetic conditions.<br><br>This test will find a large number of genetic changes. However, the majority of changes will not be reported. These include changes for which there is not enough information to know what they mean. It may be years before we understand what most of the differences in our genes mean. It may also be years before we can find a genetic difference that causes your or your child's condition.<br><br>If we do find a genetic difference that we think caused your or your child's condition, this information usually cannot predict how serious the condition will be or at what age a person may show signs. Finding a genetic difference that explains a condition will rarely lead to a cure and may not change your or your child's treatment.
Cost associated with testing	Your health insurance may or may not cover the cost of this test. You will need to pay any portion of this test that is not covered by your health insurance. You may also have medical visits to follow up on the results of this test. Insurance coverage for such tests will

	be subject to current medical practice and your insurance policy coverage. If you seek insurance coverage for this test, you may be required to release your/your child's results to your health insurance company for payment purposes.<br><br>Further testing of you and/or your family may be needed to confirm your test results, which could result in additional expense to you.
Types of results that might be returned	The goal of this test is to find the genetic cause(s) for your or your family member's medical condition. If a genetic cause is identified, it may change how your doctor cares for you. It may also allow your family members to learn about the chances that the condition will affect them. However, this test may also find genetic differences where we are not sure if they relate to your medical condition. We call these differences "variants of uncertain significance." Alternatively, it is possible that this test will not find the genetic cause of your medical condition. In fact, most patients do not receive a genetic diagnosis from this test. Please ask your healthcare provider what the estimated chance is to find the cause in your specific situation.<br><br>A small number of patients (less than 5 in 100, or 5%) who have this test will learn that they have an unexpected condition that is not related to the reason they had this test. These types of unexpected conditions often start when you are an adult, such as cancer or heart problems. When these conditions have a treatment or action available (such as going to the doctor more regularly, getting screened earlier or more often, or even surgery to lower the chance of a certain cancer), they are called "medically actionable findings" or "secondary findings." You can decide if you want to know about secondary findings or not. Incidental findings are medically actionable findings that are discovered during the course of analysis to try to find the genetic cause of your/your child's condition. [Insert language per the laboratory's policy on how incidental findings are handled/reported and whether opt out is an option.] Please use the test requisition form to indicate your preference for receiving these types of findings.
Method and timing of result delivery	The test usually takes 8-12 weeks to complete. The laboratory will make every effort to get the results to your healthcare provider as quickly as possible after receiving your sample.<br><br>You will learn the results of this test from: _________________________. This healthcare provider will receive

	a laboratory report that discusses any results. Your/your child's healthcare provider will share this information with you, and a genetic counselor may assist in explaining the results. The report containing your/your child's test results as well as any updates to those results will become part of your/your child's permanent electronic medical record and be made available to any healthcare provider treating you now or in the future.
Lab policy for sample storage and research use	After the test is done, there may be DNA remaining. The DNA sample with your name on it will be stored for at least two years at the laboratory.<br><br>The laboratory may use remaining DNA to do quality control testing and/or to develop new and better tests. Your sample would be de-identified before use in this way.
Lab policy for data storage	Your genomic testing results will be given to the doctor who ordered it and put in your medical record. Your genomic data and how it was analyzed will be saved for at least two years after testing is completed. This way, your doctors can ask the lab to re-examine your genes if this test hasn't yet found a cause for your medical condition. The laboratory will not initially deposit your complete genetic sequence into your/your child's medical record. However, it is possible that this policy will change in the future in which case your/your child's complete genetic sequence may be incorporated into your/your child's permanent medical records. You can ask for your genomic sequencing data with a request in writing. You may have to pay a small fee to receive it. If you wish to share your data with other doctors or researchers, you may do so at that time.
Lab policy for data sharing and research studies	We may share your result with other members of the medical and scientific community, including freely available genetic databases such as ClinVar and GeneMatcher. This will be done in a way that removes your identity. This type of 'de-identified' sharing helps to improve how medical professionals understand the meaning of genetic differences. It may also help us to more accurately interpret your genetic information. It is possible, though unlikely, that you could be identified through this information.<br><br>Please initial below to tell us whether we can contact you to let you know about additional research studies in which you/your child may

	be able to participate. These research studies may include:<br><br>● A request for additional clinical records about your condition<br>● Studies to find new causes for your condition<br>● Studies to evaluate newly developed treatments for your condition<br><br>Please check one option:<br><br>______ Yes, you can contact me ______ (patient initials)<br><br>______ No, please do not contact me ______ (patient initials)
Next steps if WGS does not find an answer	What we know about genetic differences changes quickly. In time, we may learn about new genes or genetic differences that help to explain your or your child's condition. In some cases we may automatically contact your doctor with new information. But most of the time this would not be automatic. You and your doctor would instead need to contact the lab to ask for re-analysis. We recommend that you keep in contact with your health care provider on an annual basis to learn of any new developments in genetics and to provide any updates to your personal or family history. You can also request that your DNA sequence information be sent to researchers who might do further research. There may be a fee associated with these options.
Documentation of informed consent	My signature below means that:<br><br>● I read and understand the information on this form<br>● The information on this form has been explained to me<br>● All my questions and concerns have been addressed by my provider<br>● I agree to provide blood or other samples for testing<br>● I understand that I can have a copy of this form after signing<br><br>Date: _____Time: _____AM/PM___________________________<br><br>Patient/Surrogate Decision Maker

	Signature<br><br>Print Name: ____<br><br>I attest that I discussed with the patient all relevant aspects of this testing, including the indications,<br><br>risks, and benefits, as compared with alternative approaches, and answered any questions.<br><br>Date: _____Time: _____AM/PM____________________________<br><br>Healthcare Provider Signature<br><br>Print Name: ____

Supplementary Discussion

Additional analysis, prioritization, classification, and reporting considerations, including targeted variant callers, risk alleles, regions of homozygosity, pharmacogenomic variation, and genes and variants of uncertain significance.

Runs of homozygosity

Genomic sequencing data can also be used to detect runs of homozygosity (ROH)[1–3]. Large ROH on a single chromosome may suggest uniparental disomy (UPD) in singleton cases[4,5]. Furthermore, when trio sequencing is performed, genomic sequencing methods are capable of detecting both isodisomy and heterodisomy[6,7]. UPD is known to contribute to the etiology of both recessive disease and imprinting disorders[8]. Alternatively, ROH across multiple chromosomes may result from consanguinity. Due to ethical issues raised by identification of consanguinity, laboratories are encouraged to develop specific policies for documenting consanguinity in accordance with existing guidelines[9].

Targeted variant callers

Targeted variant callers provide an opportunity to detect and report clinically relevant regions that are traditionally difficult to sequence (e.g. regions of homology, known pseudogenes and short tandem repeats). SMN1 copy number calling methods have been used to identify both carrier and affected status for Spinal Muscular Atrophy (SMA) [10]. Another example of a specialized variant caller is Cyrius, which enables CNV calling in CYP2D6 for pharmacogenomics reporting[11]. As computational methods improve, targeted callers may become available for other known genes and regions of clinical significance (e.g. *CYP21A2*, *PMS2*, etc.), and may be validated and incorporated into a laboratory's WGS test.

Risk Alleles and Polygenic Risk Scores

In addition to variants causative for highly-penetrant Mendelian disease, laboratories are also likely to encounter low-penetrance variants found within monogenic disease genes as well as risk alleles from genome-wide association studies (GWAS), which can be incorporated into polygenic risk scores (PRS). In some cases, genetic risk may contribute to an indication for testing in WGS. A definition of these types of results has been developed by ClinGen https://clinicalgenome.org/working-groups/low-penetrance-risk-allele-working-group/) and further guidance for clinical laboratories is under development. This guidance is needed because reporting policies for these types of variants are variable between laboratories, and decisions are often handled on an ad hoc basis depending on the professional opinions of those signing out cases.

If risk alleles are considered for reporting, it is important to note that risk and low penetrance variants may not be comprehensively assessed by variant filtering approaches that are designed to analyze highly penetrant, rare Mendelian disorders. For instance, low population frequency cutoffs may eliminate higher frequency risk alleles. Furthermore, inconsistency in the

classification terms used for these types of variants may interfere with the performance of variant database searches. As a result, ad-hoc assessment of risk variants may require specialized filtration criteria. Alternatively, laboratories may choose to limit risk reporting to a pre-curated list of variants.

The laboratory report should indicate that a risk allele or PRS does not explain a Mendelian inherited condition, particularly if no other cause of disease was identified. These results may help to explain disease penetrance[12]. They may also prove useful in the stratification of a population to manage complex traits[13–15]. We recommend that laboratories define which low penetrant variants, risk alleles and PRS may be included in a report. Laboratories should provide information to help clinicians and patients understand the limitations of these variants[16].

Pharmacogenomic variants

Variation that alters drug metabolism, known as pharmacogenomic (PGx) variants, are included in WGS analysis from some laboratories. Although PGx variants are generally not reported in the context of monogenic disease diagnostic analysis, and no primary PGx genes are currently included in the ACMG secondary findings recommendations, PGx results may be useful and relevant when performing diagnostic testing. For example, some panels for colon cancer testing also include content related to the drugs commonly used to treat colon cancer. It may therefore be useful to include PGx results on WGS reports for these indications. In general, we recommend following practices developed for panel testing to guide the minimal requirements when adding PGx content to common indications for testing. A lab may also choose to develop a panel of commonly ordered PGx markers as a separate orderable panel that can be included as a separate option for secondary findings. If a lab chooses to report these variants, it is recommended that laboratories understand guidelines from the Clinical Pharmacogenetics Implementation Consortium (CPIC; https://cpicpgx.org/guidelines/) and PharmGKB (https://www.pharmgkb.org/), and any relevant regulatory policies (https://www.fda.gov/medical-devices/precision-medicine/table-pharmacogenetic-associations).

Variants and genes of uncertain significance

Variants in known disease genes may be of uncertain significance when there is limited evidence to support a functional impact. Alternatively, the significance of a predicted disruptive variant may be uncertain if the affected gene does not have an established role in human disease[17] (https://clinicalgenome.org/curation-activities/gene-disease-validity/). In general, efforts should be made to reduce the number of VUS on WGS reports. However, clinicians may choose to initiate surveillance, undertake nonmolecular testing to confirm or exclude the disease in question, or otherwise change management based on the presence of a compelling VUS. Furthermore, alerting clinicians and patients to the presence of a potentially relevant VUS empowers them to take a more active role in seeking further information, such as signing up for alerts on ClinVar's website (https://www.ncbi.nlm.nih.gov/clinvar/docs/alerts/) or requesting future reanalysis of the variant, which may ultimately enable its reclassification. We recommend that laboratories weigh the variant-level evidence as well as the evidence supporting a connection between the patient's phenotype and the gene in order to assess the "reportability"

of each variant for that specific patient (Figure 3). Following this framework, most participating laboratories report a typical range of 0-1 VUSs per patient (Supplementary Data 6).

Variants unrelated to the primary indication for testing

We support the return of WGS findings that are likely to explain any proband phenotype provided to the laboratory, including phenotypes that were not the primary indication for testing. However, reporting pathogenic variants relevant to a documented family history of disease that is not known to be present in the tested individual can be challenging, especially if the individual does not wish to receive secondary or incidental findings. To facilitate this decision-making process, we recommend that the laboratory establish a framework and approach for the evaluation of returnable findings relevant to the patient phenotype or family history, especially if the laboratory is using EMR data not explicitly provided by the ordering clinician. Existing guidelines[18,19] are useful in this context.

Reporting decisions regarding other disease-causing variants should follow the lab's incidental and secondary findings reporting policies. Laboratories are advised to consult professional guidelines[18–20] in addition to considerations for genetic screening tests[21–23] when developing these reporting policies.

Report Readability

With increasing efforts to mainstream genomic testing and given the limited availability of genetic specialists, genetic testing, including WGS, will be ordered by a growing number of non-genetic providers. In addition, patients are taking an increasingly active role in their own healthcare and engagement with genomic information. As a result, when designing WGS reports, laboratories should also consider readability and provide connections to additional educational resources.

Treatable Genetic Conditions

The current ACMG next generation sequencing technical standard states "When reporting a gene associated with a treatable genetic disorder the laboratory should consider the addition of a reference to the treatment in the report."[18,19] Given the broad spectrum of genetic disorders detectable by WGS, we acknowledge that comprehensive reporting of available treatment options is challenging for laboratories. Emerging resources dedicated to compiling such information[24] may help to facilitate this process.

Supplementary Figure 1

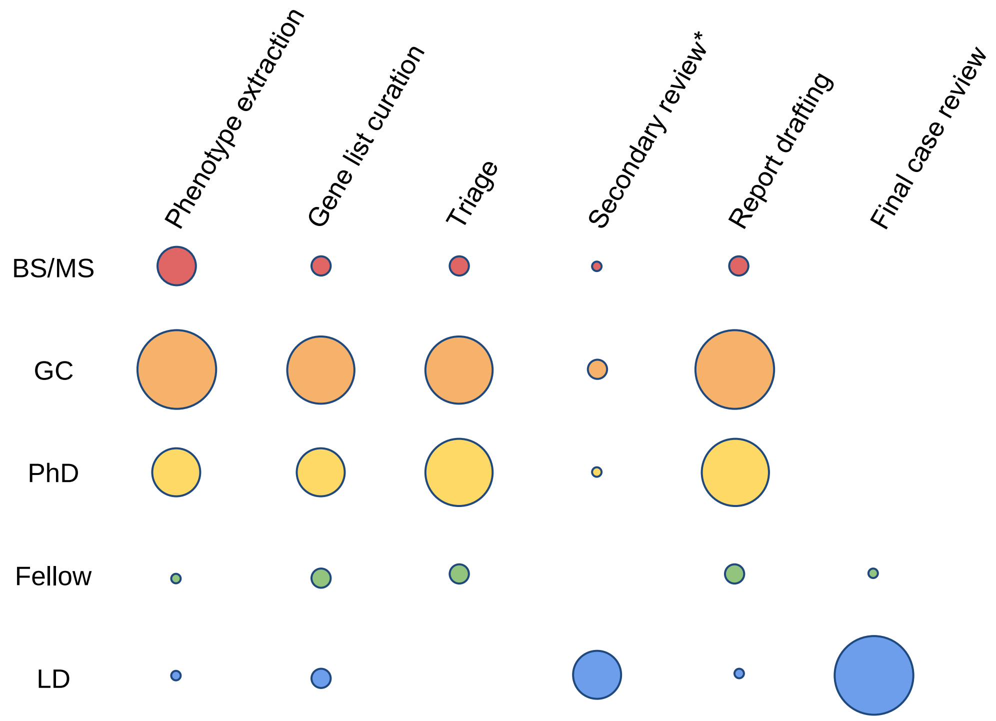

Supplementary Figure 1. Data was collected from 6 participating MGI laboratories regarding the types of staff members that have significant involvement in multiple steps of the tertiary analysis process. The size of the circle reflects the number of labs that engage each type of staff member in the indicated analysis step (e.g. 6 labs reported that genetic counselors are involved in phenotype extraction, while only 1 lab involved laboratory directors in that step). *Note that several labs report they don't have a distinct secondary review step. BS/MS - Bachelors/masters level staff; GC - Genetic counselor; PhD - PhD-level analyst, not board certified; Fellow - PhD-level ABMGG fellow/MGP resident in training; LD - Board-certified clinical laboratory director.

Supplementary References

1. Magi, A. *et al.* H3M2: detection of runs of homozygosity from whole-exome sequencing data. *Bioinformatics* **30**, 2852–2859 (2014).
2. Narasimhan, V. *et al.* BCFtools/RoH: a hidden Markov model approach for detecting autozygosity from next-generation sequencing data. *Bioinformatics* **32**, 1749–1751 (2016).
3. Ceballos, F. C., Hazelhurst, S. & Ramsay, M. Assessing runs of Homozygosity: a comparison of SNP Array and whole genome sequence low coverage data. *BMC Genomics* **19**, 106 (2018).
4. Hoppman, N., Rumilla, K., Lauer, E., Kearney, H. & Thorland, E. Patterns of homozygosity in patients with uniparental disomy: detection rate and suggested reporting thresholds for SNP microarrays. *Genet. Med.* **20**, 1522–1527 (2018).
5. Kearney, H. M., Kearney, J. B. & Conlin, L. K. Diagnostic implications of excessive homozygosity detected by SNP-based microarrays: consanguinity, uniparental disomy, and recessive single-gene mutations. *Clin. Lab. Med.* **31**, 595–613, ix (2011).
6. King, D. A. *et al.* A novel method for detecting uniparental disomy from trio genotypes identifies a significant excess in children with developmental disorders. *Genome Res.* **24**, 673–687 (2014).
7. Bis, D. M. *et al.* Uniparental disomy determined by whole-exome sequencing in a spectrum of rare motoneuron diseases and ataxias. *Mol Genet Genomic Med* **5**, 280–286 (2017).
8. Robinson, W. P. Mechanisms leading to uniparental disomy and their clinical consequences. *Bioessays* **22**, 452–459 (2000).
9. Rehder, C. W. *et al.* American College of Medical Genetics and Genomics: standards and guidelines for documenting suspected consanguinity as an incidental finding of genomic testing. *Genet. Med.* **15**, 150–152 (2013).
10. Chen, X. *et al.* Spinal muscular atrophy diagnosis and carrier screening from genome sequencing data. *Genet. Med.* **22**, 945–953 (2020).

11. Chen, X. *et al.* Cyrius: accurate CYP2D6 genotyping using whole-genome sequencing data. *Pharmacogenomics J.* **21**, 251–261 (2021).

12. Fahed, A. C. *et al.* Polygenic background modifies penetrance of monogenic variants for tier 1 genomic conditions. *Nat. Commun.* **11**, 3635 (2020).

13. Khera, A. V. *et al.* Genetic Risk, Adherence to a Healthy Lifestyle, and Coronary Disease. *N. Engl. J. Med.* **375**, 2349–2358 (2016).

14. Mega, J. L. *et al.* Genetic risk, coronary heart disease events, and the clinical benefit of statin therapy: an analysis of primary and secondary prevention trials. *Lancet* **385**, 2264–2271 (2015).

15. Khera, A. V. *et al.* Genome-wide polygenic scores for common diseases identify individuals with risk equivalent to monogenic mutations. *Nat. Genet.* **50**, 1219–1224 (2018).

16. Duncan, L. *et al.* Analysis of polygenic risk score usage and performance in diverse human populations. *Nat. Commun.* **10**, 3328 (2019).

17. Strande, N. T. *et al.* Evaluating the Clinical Validity of Gene-Disease Associations: An Evidence-Based Framework Developed by the Clinical Genome Resource. *Am. J. Hum. Genet.* **100**, 895–906 (2017).

18. Rehder, C. *et al.* Next-generation sequencing for constitutional variants in the clinical laboratory, 2021 revision: a technical standard of the American College of Medical Genetics and Genomics (ACMG). *Genet. Med.* 1–17 (2021).

19. Miller, D. T. *et al.* Recommendations for reporting of secondary findings in clinical exome and genome sequencing, 2021 update: a policy statement of the American College of Medical Genetics and Genomics (ACMG). *Genet. Med.* 1–8 (2021).

20. Kalia, S. S. *et al.* Recommendations for reporting of secondary findings in clinical exome and genome sequencing, 2016 update (ACMG SF v2.0): a policy statement of the American College of Medical Genetics and Genomics. *Genet. Med.* **19**, 249–255 (2017).

21. Hagenkord, J. *et al.* Design and Reporting Considerations for Genetic Screening Tests. *J.*

Mol. Diagn. **22**, 599–609 (2020).

22. Biesecker, L. G. Genomic screening and genomic diagnostic testing-two very different kettles of fish. *Genome Med.* **11**, 75 (2019).
23. Brothers, K. B., Vassy, J. L. & Green, R. C. Reconciling Opportunistic and Population Screening in Clinical Genomics. *Mayo Clin. Proc.* **94**, 103–109 (2019).
24. Bick, D. *et al.* An online compendium of treatable genetic disorders. *Am. J. Med. Genet. C Semin. Med. Genet.* (2020) doi:10.1002/ajmg.c.31874.
